# Transcriptomic and Ultrastructural Analyses of *Pyricularia Oryzae* Treated With Fungicidal Peptaibol Analogs of *Trichoderma* Trichogin

**DOI:** 10.3389/fmicb.2021.753202

**Published:** 2021-10-14

**Authors:** Luca Sella, Rakshita Govind, Rocco Caracciolo, Alessandra Quarantin, Van V. Vu, Silvio Tundo, Hung Minh Nguyen, Francesco Favaron, Rita Musetti, Marta De Zotti

**Affiliations:** ^1^Department of Land, Environment, Agriculture and Forestry (TESAF), University of Padova, Legnaro, Italy; ^2^NTT Hi-Tech Institute, Nguyen Tat Thanh University, Ho Chi Minh City, Vietnam; ^3^Center for Molecular Biology, College of Medicine and Pharmacy, Duy Tan University, Da Nang, Vietnam; ^4^Department of Agricultural, Food, Environmental and Animal Sciences, University of Udine, Udine, Italy; ^5^Department of Chemistry (DISC), University of Padova, Padua, Italy

**Keywords:** *Pyricularia oryzae*, antimicrobial peptides, trichogin GA IV, RNA-seq analysis, bio-pesticides

## Abstract

Eco-friendly analogs of Trichogin GA IV, a short peptaibol produced by *Trichoderma longibrachiatum*, were assayed against *Pyricularia oryzae*, the causal agent of rice blast disease. *In vitro* and *in vivo* screenings allowed us to identify six peptides able to reduce by about 70% rice blast symptoms. One of the most active peptides was selected for further studies. Microscopy analyses highlighted that the treated fungal spores could not germinate and the fluorescein-labeled peptide localized on the spore cell wall and in the agglutinated cytoplasm. Transcriptomic analysis was carried out on *P. oryzae* mycelium 3 h after the peptide treatment. We identified 1,410 differentially expressed genes, two-thirds of which upregulated. Among these, we found genes involved in oxidative stress response, detoxification, autophagic cell death, cell wall biogenesis, degradation and remodeling, melanin and fatty acid biosynthesis, and ion efflux transporters. Molecular data suggest that the trichogin analogs cause cell wall and membrane damages and induce autophagic cell death. Ultrastructure observations on treated conidia and hyphae confirmed the molecular data. In conclusion, these selected peptides seem to be promising alternative molecules for developing effective bio-pesticides able to control rice blast disease.

## Introduction

Rice (*Oryza sativa* L.) represents a staple food and one of the primary sources of nutrients for a large part of the world’s population, especially for those living in Asia (McKevith, 2004), and is the second cereal crop produced worldwide after wheat (FAOSTAT, 2020). By mid of the twenty-first century, the world’s growing population is estimated to cross 9 billion (Malik et al., 2018), which will demand double the production of cereal crops (Tester and Langridge, 2010; Nalley et al., 2016). However, many pests and pathogens can infect rice, causing significant annual losses worldwide. In particular, the most serious and economically significant disease affecting cultivated rice worldwide is the blast disease (Wilson and Talbot, 2009), caused by the hemibiotrophic fungal pathogen *Pyricularia oryzae* Cavara (anamorph of *Magnapothe oryzae* B. Couch sp. nov.). The disease is widespread in temperate rice-growing regions (Yan and Talbot, 2016; Osés-Ruiz and Talbot, 2017) and is distributed worldwide in more than 85 South Asia, European, and American countries (Martin-Urdiroz et al., 2016). Every year, approximately 10-30% of rice production is lost globally because of rice blast (Talbot, 2003), which would be enough to feed up to 60 million people (Paudel et al., 2019). Furthermore, it is predicted that climatic changes would increase (5-20%) the number of infections throughout Europe by 2030 (Bregaglio et al., 2013).

*Pyricularia oryzae* attacks rice plants at all stages of development and can infect leaves, stems, nodes and panicles (Wilson and Talbot, 2009). The infection begins when three-cell conidia land and stick to the hydrophobic waxy surface of the rice leaves. Within a few hours, conidia germinate in the presence of water, and the short germ tube differentiates at the tip by forming the appressorium, a specialized infection structure (Wilson and Talbot, 2009; Kuroki et al., 2017), while the conidium collapses by programmed cell death (PCD) (Wilson and Talbot, 2009). The appressorium is a dome-shaped structure able to generate enormous cellular turgor pressure (up to 8 MPa or 80 bars) by accumulating up to 3 M of glycerol (Foster et al., 2017), thus enabling the penetration peg to break the plant cuticle allowing the fungal invasion of the underlying epidermal cells (Wilson and Talbot, 2009). A cell wall rich in chitin and a thick layer of melanin on the inner side ensures a rapid and continual influx of water (Foster et al., 2017) and the generation of the high turgor pressure (Martin-Urdiroz et al., 2016).

At 96 h after penetration, the fungus usually switches to its necrotic phase (Yan and Talbot, 2016), and the first visible symptoms become apparent. Infections on leaves cause typical leaf spots, eye-shaped symptoms characterized by spreading lesions with necrotic center and chlorotic margin (Wilson and Talbot, 2009).

Hence, given the need to reduce reliance on synthetic fungicides, it becomes more crucial to investigate and develop new effective and sustainable alternative methods for rice blast disease management.

Using microorganisms or plant extracts with antimicrobial activity is considered a possible alternative and sustainable approach to controlling fungal diseases. For instance, several *Trichoderma* species such as *T. harzianum*, *T. virens* and *T. viride* are present as biological control agents (BCAs) in commercial fungicidal preparations registered to control several fungal pathogens (Shi et al., 2012). However, BCAs could have some limits in open field applications related to weather conditions, environment and formulation, affecting their efficacy against crop pathogens.

Due to these drawbacks, effective control of rice blast with BCAs still represents a substantial challenge, and the development of innovative strategies based on new non-toxic and eco-friendly molecules is highly required for sustainable rice protection. One possibility is to conjugate some modes of action such as antibiosis, the induction of systemic resistance and fungicidal activity of BCAs in new synthetic fungicides. Secondary metabolites of BCAs (Shi et al., 2012; Zhao et al., 2018) may be a source of new active substances. With this approach, the health hazards (Harman et al., 2004; recently highlighted by EFSA Panel on Biological Hazards, 2016) connected with the use of living fungi as BCAs and the distribution of useless products in the environment would be drastically reduced.

Therefore, the present study has been designed to test the possible use of small antimicrobial peptides (AMPs), called “peptaibols,” as biopesticides to control rice blast disease caused by *P. oryzae*. Recently, water-soluble analogs of the *T. longibrachiatum* short-length peptaibol trichogin GA IV (Szekeres et al., 2005) have been synthesized using a versatile synthetic strategy designed to reduce the impact on the environment (De Zotti et al., 2020). Three of these analogs completely inhibited the growth of *Botrytis cinerea in vitro* at low micromolar concentrations, and the most effective peptides significantly reduced gray mold symptoms on common bean, grapevine leaves and ripe grape berries (De Zotti et al., 2020).

A detailed understanding of peptides’ antimicrobial mode of action is fundamental to exploit them in field applications and improve peptide design and optimize their distribution. Indeed, although antimicrobial peptides are usually proposed to act via plasma membrane permeabilization, leading to membrane rupture and rapid lysis of microbial cells, some of them seem to be able to interact with intracellular specific targets (Wang et al., 2017).

In the present work, we first tested the *in vitro* fungicidal activity of water-soluble analogs of trichogin against four *P. oryzae* strains of different geographical origins. As previously reported (De Zotti et al., 2020), some analogs were synthesized by replacing one or more glycine residues on the hydrophilic face of trichogin by lysine, thus strengthening the amphiphilic nature of the trichogin structure; one analog was synthesized by replacing a glycine residue with an α-aminoisobutyric acid (Aib) residue, known to be a strong helix-inducer (Crisma et al., 2005); one further analog was synthesized by replacing Aib at position 8 with the cationic, C^α^ -tetrasubstituted 4-aminopiperidine-4-carboxylic acid (Api) residue to verify the effect of perturbing the hydrophobic face of the helix.

The peptides verified as most effective *in vitro* were then tested *in vivo* against one of the strains isolated from North Italy rice fields, whose virulence had been previously characterized (Roumen et al., 1997). Treatment and inoculation were performed on rice and barley. Finally, to characterize the effects of the trichogin analogs on *P. oryzae* cells and improve knowledge on their mode of action, we analyzed the transcriptome by RNA-seq and the ultrastructure of the fungus after treatment with one of the most effective peptides.

## Materials and Methods

### Fungal Strains, Growth Conditions, and Conidia Production

Four *Pyricularia oryzae* strains of different geographical origins (IT10 and AP isolated from North Italy, Guy11 from French Guiana, and I203 from Vietnam) were used in this study. Fungal strains were first cultivated on Potato Dextrose Agar (PDA; Difco Laboratories) incubating plates at 25°C for 7 days. Conidia were obtained by inoculating 7 day-old mycelia plugs on Oat Meal Agar (OMA) plates prepared with a protocol modified from Gooding and Lucas (1959). Briefly, 100 g of oat flakes were dissolved in 1 L of deionized water at 70°C for 1 h under continuous stirring, filtered through cheesecloth, and subsequently autoclaved. After 12 days of incubation at 28°C and 16/8 h light/dark conditions (Hosseyni-Moghaddam and Soltani, 2013), conidia were collected by adding 4 mL of water supplemented with 0.025% (v/v) Tween 20 into each 9-mm OMA plate and by gently scraping with a sterile loop. The suspension was filtered through 100 μm nylon sterile filters (Corning cell strainer, USA) and conidia were counted with a Thoma cell chamber (Fein-Optik, Bad Blankenburg, Germany) and adjusted to obtain a final concentration of 5 × 10^5^ per mL. Conidia were finally stored at −80°C.

### Peptide Analogs Design and Synthesis

Eleven water-soluble analogs of trichogin GA IV from *Trichoderma longibrachiatum* (De Zotti et al., 2009) were designed as reported in De Zotti et al. (2020). Five shorter cost-effective analogs were also produced. Pep 4Rink has also been conjugated with the Fluorescein Isothiocyanate (FITC) fluorophore. The conjugation to FITC was performed at the peptide C-terminus, since that position affects the properties of the peptide only marginally, as reported in Dalzini et al. (2016). The list of the peptide analogs and their aminoacidic sequence are reported in Table 1.

**TABLE 1:** List of peptide analogs used in this work and their amino acidic sequence.

Trichogin GA IV	*n*Oct-Aib-Gly-Leu-Aib-Gly-Gly-Leu-Aib-Gly-Ile-Lol
Pep 2 (K2)	*n*Oct-Aib-**Lys**-Leu-Aib-Gly-Gly-Leu-Aib-Gly-Ile-Lol
Pep 2Rink (K2)	*n*Oct-Aib-**Lys**-Leu-Aib-Gly-Gly-Leu-Aib-Gly-Ile-Leu-NH2
Pep 3 (K5)	*n*Oct-Aib-Gly-Leu-Aib-**Lys**-Gly-Leu-Aib-Gly-Ile-Lol
Pep 4 (K56)	*n*Oct-Aib-Gly-Leu-Aib-**Lys**-**Lys**-Leu-Aib-Gly-Ile-Lol
Pep 4Rink (K56)	nOct-Aib-Gly-Leu-Aib-**Lys**-**Lys**-Leu-Aib-Gly-Ile-Leu-NH2
Pep 4C (K56)	nOct-Aib—**Lys**-**Lys**-Leu-Aib-Gly-Ile-Lol
Pep 4C1 (K56)	nOct-Aib-Gly-Leu-Aib-**Lys**-**Lys**-Leu—Leu-NH2
Pep 4C2 (K56)	nOct-Aib—**Lys**-**Lys**-Leu-Aib-Gly-Ile-Leu-NH2
Pep 5 (K259G6)	*n*Oct-Aib-**Lys**-Leu-Aib-**Lys**-Gly-Leu-Aib-**Lys**-Ile-Lol
Pep 6 (K5U6)	*n*Oct-Aib-Gly-Leu-Aib-**Lys**-**Aib**-Leu-Aib-Gly-Ile-Lol
Pep 7 (K26)	*n*Oct-Aib-**Lys**-Leu-Aib-Gly-**Lys**-Leu-Aib-Gly-Ile-Lol
Pep 8 (ApiC)	*n*Oct-Aib-Gly-Leu-Aib-Gly-Gly-Leu-**Api**-Gly-Ile-Lol
Pep 12 (HPA3NT3)	H-Phe-**Lys**-Arg-Aib-**Lys**-**Lys**-Aib-Phe-**Lys**-**Lys**-Aib-Trp-Asn-Trp-**Lys**-NH2
Pep 19	Palmitoyl-His-Ala-Ala-His-**Lys**(ε-Ser)-Gly-COOH
Pep 22 (K6)	*n*Oct-Aib-Gly-Leu-Aib-Gly-**Lys**-Leu-Aib-Gly-Ile-Lol
Pep 22Rink (K6)	nOct-Aib-Gly-Leu-Aib-Gly-**Lys**-Leu-Aib-Gly-Ile-Leu-NH2

Peptides were synthesized by manual or semiautomatic (Biotage MultiSynTech) solid-phase synthesis (SPPS) as reported in De Zotti et al. (2020). All crude peptides were obtained with a degree of purity higher than 85%, purified to 95-99% by medium-pressure liquid chromatography (Isolera Prime, Biotage) (De Zotti et al., 2020) and freeze-dried.

### *In vitro* Screening of the Antimicrobial Activity of Peptaibols

The activity of 16 different analogs of Trichogin GA IV (Table 1) against *P. oryzae* strains was evaluated by an *in vitro* assay. Microtiter plate wells were filled with 200 μl of Potato Dextrose Broth (PDB; Difco Laboratories) at pH 6.85 containing 1 × 10^5^ conidia per mL. The peptides were dissolved in water and assayed at 25°μM or 50°μM. Samples without the peptide were used as control. After incubating in the dark at 25°C for 96 h, fungal growth was evaluated by measuring the absorbance at 450 nm (Granade et al., 1985) in three wells per thesis. The experiment was replicated three times.

### Plant Growth

Barley (cv. Alora) and rice (cv. Vialone Nano) seeds were germinated in Petri dishes containing three sterile filter papers, moistened with 10 mL of sterile water, and incubated for 3 days at 25°C in the dark.

Barley and rice seedlings were transplanted into 10 × 5 cm plastic pots (four seedlings per pot) containing universal soil (acid peat, simple non-composted vegetable amendment, mineral fertilizer, pH 6) and plantlets were grown in a climatic chamber with 14 h photoperiod at 22/20°C day/night temperature for 6 and 8 days, respectively.

### Peptide Treatment and Fungal Inoculation on Barley and Rice Leaves

The first leaf of each 6-day-old barley seedling was cut and placed in a Petri dish (14-cm diameter) containing three filter papers moistened with 10 mL of sterile water. Basal and apical parts of the leaves were fixed with glass slides.

Eight-day-old rice seedlings were horizontally placed on a tray containing sterile wet paper, and their roots were covered with sterile wet tissue paper.

Two-point inoculations per barley and rice leaf were performed by dropping 10 μL of a suspension containing the peptide at 50 μM, 1 × 10^3^ conidia of *P. oryzae* IT10 strain and 10% (v/v) Tween 20. For each peptide, at least five leaves were inoculated in each biological replicate and at least three independent biological replicates were performed. Fungal conidia suspended in water were used as a positive control. Petri dishes and trays were sealed with a transparent polyethylene film, and were incubated at 25°C under 16/8 h light/dark conditions.

After 7 days of incubation, the lesion area on barley leaves was calculated by the Assess© Software (American Phytopathological Society – APS). In rice, the severity of blast symptoms was estimated after 8 days by identifying 5 levels of disease severity (DS0-5) (Supplementary Figure 1) and a Disease Severity Index (DSI) was calculated as reported in Pak et al. (2017). The percentage of reduction of disease severity in comparison to the control not treated with the peptide was calculated as follows: (DSIcontrol - DSItreatment/DSIcontrol)×100.

### Statistical Analysis for *in vitro* and *in vivo* Assays

At least three independent experiments were performed for all in vitro and in vivo assays. Data were statistically analyzed by applying one-way ANOVA followed by Bonferroni–Holm test.

### Light Microscopy of Peptide Treated Fungal Conidia

Ten microliters of spore suspension containing 1 × 10^5^ conidia mL^–1^ of *P. oryzae* (IT10 strain) untreated or treated with 50 μM of Pep 2, 4, 4rink, 4C, 5, 6, 8, 19 and 22 were incubated at 25°C and observed under a light microscope (Leica DM750; Leica Microsystems, Germany) up to 48 h.

Fungal conidia were also treated with Pep 4Rink conjugated with the FITC fluorophore, incubated at 25°C and observed up to 48 h under a light fluorescence microscope (Leica DM4000 B, Leica Microsystems, Germany). Preliminary *in vitro* assays showed that the inhibitory activity of the peptide conjugated with the fluorophore was similar to that of the non-conjugated peptide.

### Transmission Electron Microscopy of Peptide-Treated Fungal Conidia and Hyphae

Transmission electron microscopy (TEM) observations were carried out to evaluate the ultrastructural changes induced in fungal cells following Pep treatment. Germinating conidia (5 × 10^5^ mL^–1^) of *P. oryzae* IT10 strain were cultured at 25°C on CM. After 12 or 24 h, samples were treated with 50 μM or 25 μM of Pep 4Rink and incubated for 16 or 24 h at 25°C in the dark. Untreated samples were also kept in the same conditions and served as controls. Conidia and hyphae were thereafter collected and fixed in 3% (v/v) glutaraldehyde in phosphate buffer (PB) 0.15 M for 2 h, at 4°C. After rinsing in PB, samples were post-fixed with 1% (w/v) OsO_4_ in 0.15 M PB for 2 h at 4°C, dehydrated with an ethanol gradient and transferred to pure propylene oxide. Samples were then embedded in Epon/Araldite epoxy resin (Electron Microscopy Sciences, Fort Washington, PA, USA). Ultra-thin sections were collected on uncoated copper grids, stained with UAR-EMS (uranyl acetate replacement stain, Electron Microscopy Sciences) and then observed under a PHILIPS CM 10 TEM (FEI, Eindhoven, The Netherlands), operated at 80 kV and equipped with a Megaview G3 CCD camera (EMSIS GmbH, Münster, Germany). Seven non-serial sections from each sample were analyzed.

### Conformational Analysis

Circular dichroism (CD) was performed on a Jasco J-715 spectropolarimeter (Tokyo, Japan), as reported in De Zotti et al. (2020), with samples containing peptides at 50 μM and 1 × 10^5^ conidia mL^–1^ of *P. oryzae* (IT10 strain). Samples were incubated for 48 h at 25°C in the dark in 10 mM phosphate buffer pH 7. We used a fused quartz cell with 1-mm path length (Hellma). The observed ellipticity values were expressed in terms of total molar ellipticity [θ]_*T*_ (deg × cm^2^/dmol).

### Transcriptomic Analysis

#### Samples Preparation and Sequencing

Conidia of *P. oryzae* (IT10 strain) were cultured in 1.8 mL Complete Medium (CM; Talbot et al., 1993) at a final concentration of 2 × 10^5^ conidia per mL for 3 days at 30°C under shaking at 150 rpm. After three days, 50 μM of Pep 4rink, one of the most effective peptides *in vivo*, were added to samples containing actively growing mycelium. Untreated samples were used as control. Three h after peptide treatment, fungal mycelia were collected, filtered with 100 μm Cell Strainer (Corning^®^), rinsed with sterile water and stored at −80°C for RNA extraction.

Total RNA was extracted with the RNeasy Plant Mini kit (Qiagen) following the manufacturer’s instructions, performing the DNase digestion directly on the column. The integrity and concentration of the RNA samples were checked with Qubit fluorimeter and Bioanalyzer. RNA samples with RIN values <7 were discarded. Four biological replicates of each treatment were submitted to RNA sequencing and analysis at CRIBI Biotechnology Center (University of Padova).

Two μg of total RNA were subjected to PolyA tail capture and rRNA depletion for selection of mRNA. Directional (stranded) RNA libraries were prepared, quantified and subjected to quality control. Sequencing was performed on Illumina Nova Seq 6000 platform. Twenty million reads were produced for each sample and checked for quality control.

#### Bioinformatic Analysis

Reads were aligned to the reference genome (*Magnaporthe oryzae* 70-15 strain). The sequencing data were analyzed with RSEM (v. 1.3.3), which provides the raw count matrix as the final output. Normalization of row counts and analysis of differentially expressed genes (DEGs) in control versus treated samples were performed with edgeR (v. 3.11). In detail, all genes that had at least 10 counts in each sample were filtered before normalization. The DEGs were selected by considering genes with adjusted *p*-value <0.01 (BH method), FDR (False Discovery Rate) < 0.05, and a log_2_FoldChange (FC) > 0.5 or < −0.5.

The principal component analysis (PCA) was also performed on the normalized count matrix.

#### Annotation of Differentially Expressed Genes

All DEGs with FDR value < 0.05 and log2(FC) > 0.5 or < -0.5 were annotated (UniProt ID and description of gene function) based on the *M. oryzae* strain 70-15 genome version and on Ensembl and XML databases obtained from UniprotKB. Genes encoding uncharacterized proteins were subjected to Pfam domain search using HMMER^1^ to identify their possible functions.

Data were exported and processed in *R* (data.matrix). Graphs have been created in *R* using the “ggplots2,” “heatmap.2,” and “edgeR” libraries.

#### Gene Ontology and FunCat Enriched Categories

Gene Ontology (GO) and Functional Catalog (Fun Cat) enrichment analyses were performed on the DEGs by using the FungiFun2 database^2^ (Priebe et al., 2015).

### Quantitative PCR

To validate the RNA-seq results, we analyzed the relative expression of 11 genes randomly selected by quantitative PCR (qPCR). RT-qPCR was performed on a Rotor-Gene Q 2plex (Qiagen) using specific primers (Supplementary Table 1) and RNA extracted as above reported from mycelia of *P. oryzae* untreated or treated for 3 h with Pep 4Rink. RT was performed as reported in Sella et al. (2016). The 20 μL reaction mixture contained 10 μL of 2X Rotor-Gene SYBR Green PCR MasterMix (Qiagen), 0.5 μM of each specific primer, and 3 μL of cDNA as template. The qPCR was performed by repeating 40 times the following cycle: 15 s at 95°C; 15 s at 55°C; 30 s at 72°C. Reactions were performed in triplicate and the experiment was repeated with two biological samples. Relative expression results were analyzed using the Rotor-Gene v. 2.0.3.2 software (Qiagen) and the REST tool (Relative Expression Software Tool; Pfaffl et al., 2002). The *P. oryzae* housekeeping genes actin (MGG_03982) and glyceraldehyde-3-phosphate dehydrogenase (MGG_01084) were used for normalization of gene expression, both housekeeping genes showing equal expression stability under the given conditions.

## Results

### *In vitro* Screening of the Antimicrobial Activity of Trichogin Analogs Against Different *P. oryzae* Strains

**FIGURE 1:**
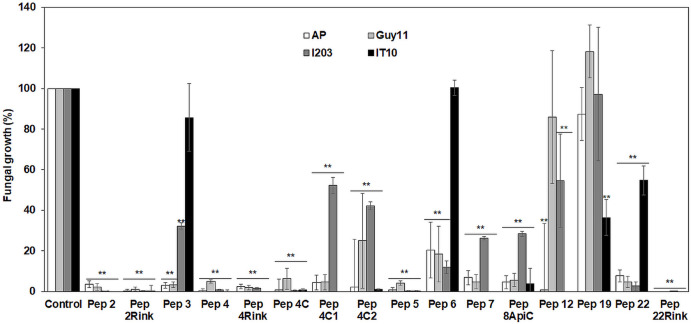
Data represent the percentage (%) of *Pyricularia oryzae* (strains AP, Guy11, I203 and IT10) mycelium growth after 96 h of incubation in the presence of 50 μM of each peptide compared to the growth of the untreated mycelium. Standard errors were indicated by error bars. Data were statistically analyzed by applying the one-way Anova Bonferroni–Holm test (**significant difference at *p* < 0.01).

A preliminary *in vitro* screening of the antimicrobial activity of the trichogin analogs was performed at 25 μM against the *P. oryzae* AP strain. Since the most effective peptides showed a maximum fungal growth reduction of about 60-75% compared to control (not shown), we decided to compare the activity of the 16 analogs against the four *P. oryzae* strains increasing the peptide concentration to 50 μM. After 96 h of incubation, compared to the untreated control, seven peptide analogs (Pep 2, 2Rink, 4, 4Rink, 4C, 5 and 22Rink) showed more than 90% inhibition of fungal growth regardless of the fungal strain (Figure 1). The remaining peptides (Pep 3, 4C1, 4C2, 6, 7, 8Api, 12, 19 and 22) showed relatively little or no inhibition against one or two strains (Figure 1). The I203 strain seemed poorly inhibited by four of these nine peptides, followed by IT10 (three peptides less active), Guy11 (two peptides less active) and AP (one peptide less active). Pep 19 did not inhibit Guy11, AP and I203 strains and partially inhibited the IT10 strain (Figure 1). Trichogin was also ineffective against *P. oryzae* (data not shown).

### Treatment of Barley Leaves With Peptide Analogs and Effect on Blast Symptoms Development

**FIGURE 2:**
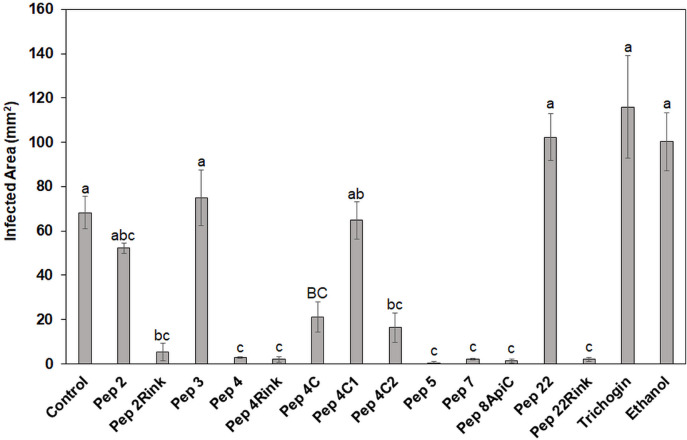
Data represent the lesion area (expressed in mm^2^) caused by *Pyricularia oryzae* IT10 strain on barley leaves (cv. Alora) at 7 days post-inoculation (dpi) and are the mean of at least three independent biological replicates. Standard errors were indicated by error bars. Significant inhibition (*p* < 0.05) of lesions was observed with Pep 2Rink, 4, 4Rink, 4C, 4C2, 5, 7, 8Api and 22Rink compared to the Control. Trichogin at 50 μM concentration, resuspended in 3% (v/v) ethanol, and 3% (v/v) ethanol were also included as negative controls. Data were statistically analyzed by applying the one-way Anova Bonferroni–Holm test (different lowercase letters indicate significant differences at *p* < 0.01, different uppercase letters indicate significant differences at *p* < 0.05).

Twelve peptide analogs totally or partially inhibiting the *P. oryzae* strains *in vitro* were tested at 50 μM to evaluate their efficacy in reducing blast symptoms. The screening was performed on barley, a secondary host of the fungus (Guo et al., 2017) that allowed to use a detached leaf assay. Barley leaves were inoculated with 1 × 10^3^ fungal spores, and seven days post inoculation, the symptomatic area was evaluated and compared with that of the untreated leaves. The experiment revealed some differences in efficacy between the peptides. In comparison with untreated barley leaves, Pep 4, 4Rink, 5, 7, 8Api and 22Rink showed a reduction of blast lesion area higher than 95%, followed by Pep 2Rink (about 92% of decrease), 4C2 and 4C (about 75 and 70% of reduction, respectively), while Pep 2, 4C1 and 22 were ineffective. The trichogin at 50 μM concentration was also ineffective (Figure 2). Representative lesions caused by *P. oryzae* on barley leaves are shown in Supplementary Figure 2. No visible toxic effects were observed on leaves treated with the peptides (not shown).

### Treatment of Rice Leaves With Peptide Analogs and Effect on Blast Symptoms Development

**FIGURE 3:**
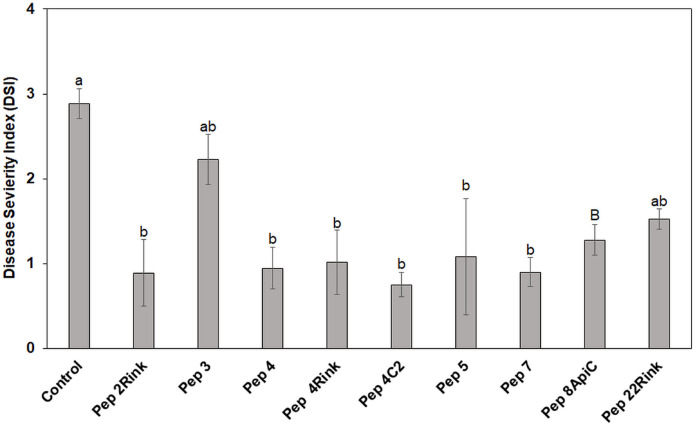
Data represent the mean Disease Severity Index (DSI) evaluated on rice (cv. Vialone Nano) at 8 days post inoculation (dpi) with spores of *Pyricularia oryzae* IT10 strain and are the mean of at least three independent biological replicates. Standard errors were indicated by error bars. Significant reduction (*p* < 0.05) in disease severity was observed with all peptides except Pep 22Rink. Pep 3 was used as a negative control, being ineffective against *P. oryzae*. Data were statistically analyzed by applying the one way-Anova Bonferroni–Holm test (different lowercase letters indicate significant differences at *p* < 0.01, different uppercase letters indicate significant differences at *p* < 0.05).

The most effective peptides in reducing blast symptoms on barley leaves were also assayed for their ability to protect leaves of rice seedlings from *P. oryzae* infection. Pep 3, which proved ineffective in protecting barley from *P. oryzae*, was also assayed. Leaves of rice seedlings (cv. Vialone nano) were treated with the peptides at 50 μM concentration and inoculated with a suspension containing 1 × 10^3^ fungal spores. After 8 days, the mean Disease Severity Index (DSI) of rice blast was calculated. On the whole, all peptides, except Pep 22Rink, determined a significant (*p* < 0.05) reduction of leaf blast symptoms compared to the untreated control. Pep 3 confirmed the inefficacy previously demonstrated on barley. The peptides 2Rink, 4, 4Rink, 4C2, 5 and 7 proved to be the most effective, with a 65-70% reduction in DSI (Figure 3), while Pep 8ApiC and 22Rink showed about 50-55% DSI reduction. Representative lesions caused by *P. oryzae* on rice leaves are shown in Supplementary Figure 3.

### Light Microscopy Analysis of *Pyricularia oryzae* Spores Treated With Peptides

**FIGURE 4:**
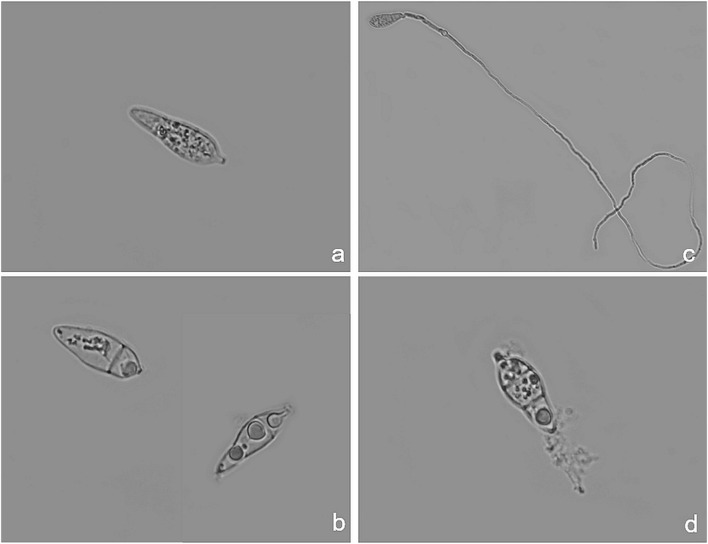
Light microscopy pictures of *Pyricularia oryzae* (IT10 strain) conidia after incubation with Pep 4Rink in Potato Dextrose Broth (PDB) medium. **(a)** untreated *P. oryzae* conidium before peptide treatment; **(b)** non-germinated *P. oryzae* conidia at 48 h after peptide treatment, showing cytoplasmic agglutination; **(c)** untreated germinated *P. oryzae* conidium at 48 h; **(d)** early germinated *P. oryzae* conidium showing lysed hypha at 48 h from peptide treatment.

After clear evidence of the inhibiting activity displayed by some peptide analogs *in vitro* and *in vivo*, light microscopy observations showed that *P. oryzae* conidia treated with the most effective peptides (Pep 2, 4, 4Rink, 4C, 5, 8Api, and 22Rink) exhibited important morphological changes. In particular, cells of the treated spores were characterized by a densely agglutinated cytoplasm separated from the rigid cell wall, possibly due to the loss of intracellular liquid (Figure 4b and Supplementary Figure 4). Sometimes, treated spores germinate, but their hyphae were early lysed (Figure 4d). Conversely, we did not notice any specific cytoplasmic alteration in the untreated spores (Figure 4a and Supplementary Figure 4). *P. oryzae* spores treated with the analogs found as ineffective *in vitro*, such as Pep 6 and 19, germinated normally and did not show any morphological alteration (Figure 4c and Supplementary Figure 4).

When observed under a fluorescent microscope, peptide-treated spores of *P. oryzae* produced auto-fluorescence, indicating that they were non-viable (Wu and Warren, 1984; Supplementary Figure 5D), while untreated and germinated spores did not develop auto-fluorescence (Supplementary Figure 5B). One of the most effective peptides, tagged with the FITC fluorophore, localized at the fungal spore cell wall and intracellularly in the densely agglutinated cytoplasm. Moreover, the spore septa did not show any fluorescence (Supplementary Figure 5F).

### Conformational Analysis of Peptides by Circular Dichroism

**FIGURE 5:**
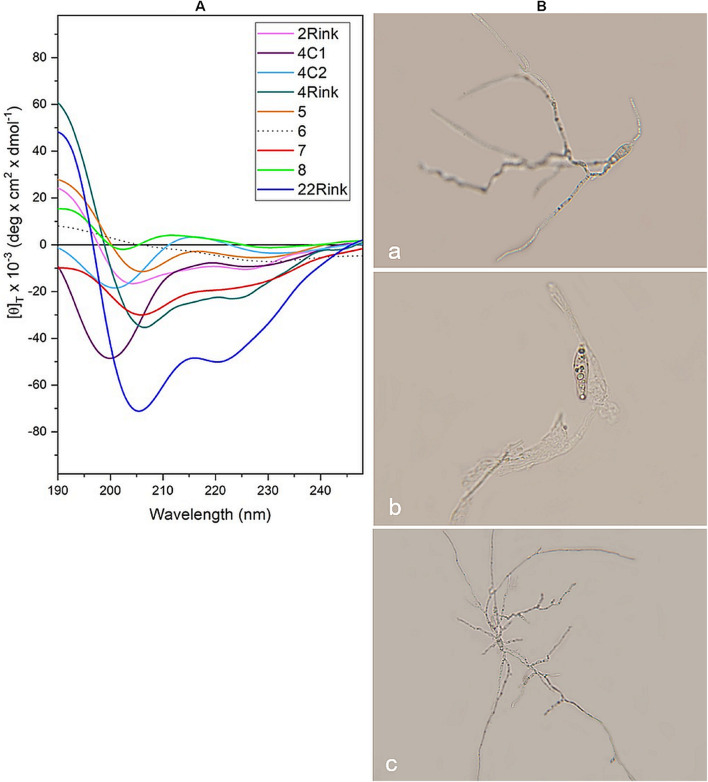
**(A)** CD spectra acquired *in vitro* for pep 2Rink, 4Rink, 4C1, 4C2, 5, 6, 7, 8Api and 22Rink at 50 μM in the presence of *Pyricularia oryzae* (IT10 strain) incubated for 48 h in phosphate buffer pH 7 at 25°C; **(B)** Optical microscopy pictures representing *P. oryzae* conidia untreated **(a)** and after 48 h of incubation with Pep 2Rink **(b)** or Pep 6 **(c)** in 10 mM phosphate buffer at pH 7. In the presence of the non-effective Pep 6, the fungal mycelium grew better than the untreated control.

Circular dichroism analysis was carried out on *P. oryzae* IT10 strain spores in the presence of eight peptides that proved active *in vitro* (Pep 2Rink, 4C1, 4C2, 4Rink, 5, 7, 8Api, 22Rink) and one inactive (Pep 6). Most peptides retain their well-developed helical conformation. Interestingly, while Pep 8ApiC spectrum seems to be altered by the presence of the fungal pathogen, we observed a loss of signal for Pep 6 (Figure 5A). Since this peptide analog was not able to effectively inhibit *P. oryzae* growth, we hypothesized that the fungus degraded it. Indeed, light microscopy observations showed that *P. oryzae* mycelia grew better in the presence of this peptide than in the untreated control (Figure 5B). CD analysis performed on the short analogs 4C2 and 4C1 showed unaltered profiles in the presence of the fungus (Figure 5A).

### RNA-Seq Analysis of *Pyricularia oryzae* Treated With Pep 4Rink

To verify the relationships among the biological replicates, the PCA was performed on the normalized count matrix of each biological replicate at 3 h. The analysis showed a homogeneous distribution of the biological replicates. As expected, the samples were arranged into two partially separated groups, thus confirming this method’s and the experiment’s reliability (Supplementary Figure 6).

#### Heatmap of Control vs. Treatment

**FIGURE 6:**
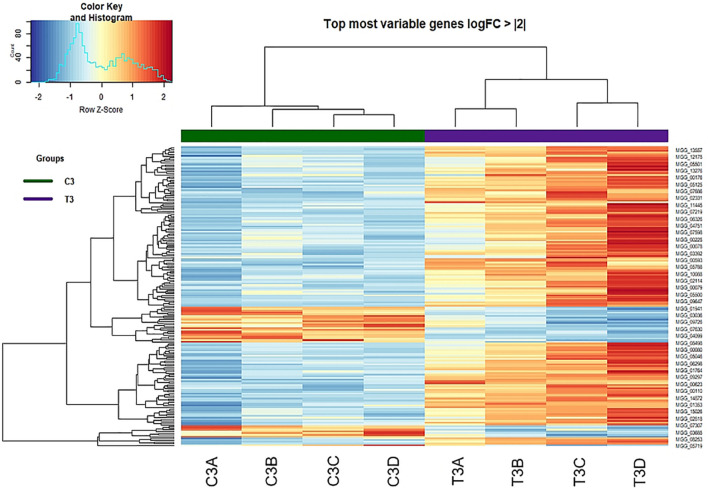
Heat-map of DEGs with log Fold Change of at least + 2 or –2 identified at 3 h from treatment (Control vs. Treatment).

A heatmap for control and peptide-treated biological replicates was designed on DEGs showing a logFold Change of at least + 2 or −2 (Figure 6). The two groups (control and treated samples) were perfectly clustered, outlining a clear profile and highlighting a higher number of upregulated genes in the treated samples.

#### Differentially Expressed Genes, Volcano Plot, and Venn Diagram

**FIGURE 7:**
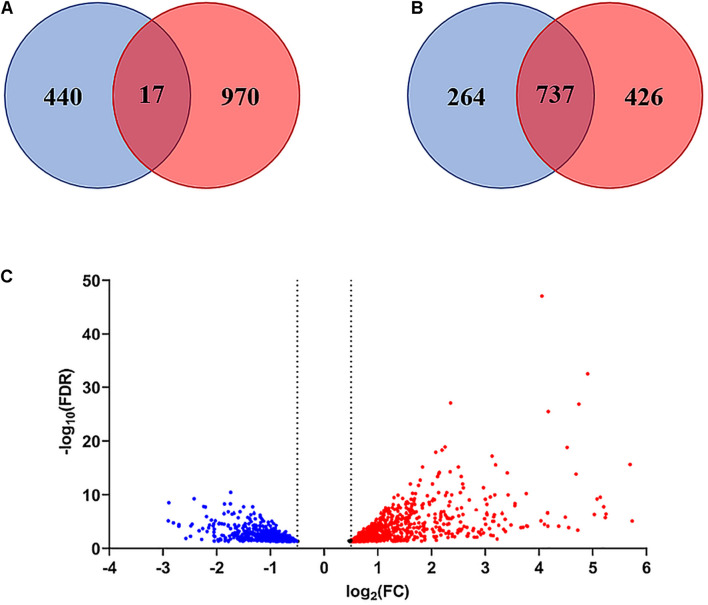
Venn diagram and Volcano plot of differentially expressed genes (DEGs). **(A)** Venn diagram of DEGs with FDR < 0.05 considering log_2_(Fold Change) > 0.5 or < –0.5 as threshold. **(B)** Venn diagram of DEGs with FDR < 0.05 considering log_2_(Fold Change) > 1or < –1 as threshold. **(C)** Volcano plot showing the distribution of DEGs with FDR < 0.05 (log_2_FC vs. –Log_10_FDR) at 3 h after treatment. Up-regulated genes are indicated in red, down-regulated genes in blue. Genes in the overlapping area of the Venn diagrams are stably expressed. FC = Fold Change.

The comparison of the control and peptide treated samples identified 1427 DEGs with FDR <0.05 and *p*-Value <0.01, including 986 upregulated and 441 downregulated genes. The Volcano plot reported in Figure 7C shows the overall distribution of DEGs. The log_2_(Fold Change) >0.5 or < −0.5 included 98.9% of DEGs with FDR < 0.05 (Figure 7A), while the log_2_FC > 1 or < −1 comprised only 48.4% of the DEGs (Figure 7B). Therefore, we chose log_2_(Fold Change) > 0.5 or < −0.5 as the threshold. The list of DEGs is reported in Supplementary Table 2.

#### Enrichment Analysis Gene Ontology and Fun Cat Categories

To highlight the general effect of peptide treatment on *P. oryzae*, we analyzed the significant DEGs by using the GO and FunCat databases.

FunCat analysis classified DEGs according to 8 main categories: cellular transport, transport facilitation, and transport routes; protein fate; biogenesis of cellular components; metabolism; protein with binding function or cofactor requirement; cell rescue, defense and virulence; biogenesis of cellular components; interaction with the environment.

The FunCat descriptions of the upregulated DEGs at 3 h were involved in proteasomal degradation, autoproteolytic and proteolytic processing (representing 9.8% of the total upregulated DEGs), vesicle formation and intracellular transport, vacuolar/lysosomal transport and endocytosis (10.6%), and stress response (5.37%) (Supplementary Table 3). The downregulated DEGs at 3 h were involved in transport facilities, antibiotic resistance, and glutathione conjugation reaction (Supplementary Table 4).

Gene ontology analysis classified DEGs based on three ontology types: Biological Process (BP), Cellular Component (CC), and Molecular function (MF).

Among the GO terms enriched in upregulated DEGs at 3 h, those more interesting were membrane composition/structure (40% of the total upregulated DEGs), vesicular transport (5.8%), Ca^2+^ transport (3.5%), proteasome complex (2.8%) and melanin biosynthesis (0.7%) (Supplementary Table 5). GO term enrichment analysis classified most of the downregulated DEGs at 3 h in three groups: oxidation-reduction process (45.6% of the total downregulated DEGs), mycelium development (23%) and membrane composition/structure (17.4%) (Supplementary Table 6).

### Functional Classification of *Pyricularia oryzae* Differentially Expressed Genes

About one-third of the *P. oryzae* DEGs were functionally annotated based on their sequence homology to other species (Mathioni et al., 2013). We have analyzed the uncharacterized DEG sequences for Pfam domains and identified the putative function of about another third of genes. Only DEGs functionally annotated with a recognized Pfam domain were further analyzed and grouped based on their putative function. The most interesting DEGs are summarized in Supplementary Tables 7, 8.

#### Oxidation-Reduction Balance and Stress Response

Several genes, such as cytochrome monooxygenases and oxidoreductases, were differentially expressed (up- or downregulated) after 3 h of the peptide treatment, thus indicating a general alteration in the oxidation-reduction balance. In particular, nine up- and five down-regulated genes encode for the cytochrome P450 monooxygenase, which plays essential roles in a wide variety of metabolic processes such as the detoxification of endogenous compounds and xenobiotics (Shin et al., 2018). Besides, 18 genes encoding proteins with an oxidoreductase domain were differentially expressed (eleven up- and seven down-regulated).

We also observed the upregulation of genes encoding galactose oxidase, isoamyl alcohol oxidase (AOX), superoxide dismutase (SOD), and Cu radical oxidase, all involved in the formation of hydrogen peroxide (Whittaker, 2005; Kersten and Cullen, 2014), as well as the downregulation of genes encoding glutathione peroxidase (GPX) and catalase (CAT), which were responsible for H_2_O_2_ reduction to water.

Interestingly, several genes dealing with glutathione, a molecule known to play key roles in response to several stresses in fungi such as oxidative stress (Pócsi et al., 2004), were differentially expressed. In particular, five genes encoding glutathione S-transferase, an enzyme involved in the detoxification of toxic substances by their conjugation with glutathione and in the detoxification of reactive oxygen species (ROS) together with SODs and catalases (Gullner et al., 2018), and eight genes encoding enzymes involved in glutathione synthesis and degradation as well as drug and xenobiotic detoxification were up or down-regulated.

Noteworthy, several endo-glucanase encoding genes, which are considered to play a general protective mechanism against stress in fungi (Mathioni et al., 2013), are upregulated after 3 h of treatment.

#### Transporters and Detoxification

The ATP-binding cassette transporter superfamily members remove many toxic compounds by coupling transport with ATP hydrolysis or a proton gradient (Coleman and Mylonakis, 2009). The up-regulation of five ABC transporter encoding genes and genes encoding flavin binding monooxygenase and ATS1 N-acetyl transferase, involved, respectively, in the oxidation of toxic xenobiotics (Gul et al., 2016) and metabolization of drugs and other xenobiotics (Karagianni et al., 2015), confirmed that the treated fungus activates general detoxification mechanisms against the stress induced by the peptide already after 3 h from treatment.

#### Autophagy and Proteasome

One of the responses activated by the fungus 3 h after peptide treatment is the up-regulation of five genes encoding autophagy-related proteins (ATG3, ATG4, ATG7, ATG9, and ATG17), two genes encoding proteins with a NACHT domain, a nucleoside triphosphate domain found to be involved in the control of heterokaryon incompatibility and in apoptosis (Bidard et al., 2013), and seven genes encoding heterokaryon incompatibility (HET) proteins, whose overexpression can result in cell death (Paoletti and Clavé, 2007).

Besides, five genes encoding for the Hsp70 chaperone were found to be early upregulated. Chaperones assist a variety of protein folding processes, suggesting that peptide treatment could cause protein misfolding. Indeed, we found as also upregulated 17 proteasome-related genes. The proteasome is a large 33- subunit protein complex, which controls the degradation of ubiquitinated intracellular proteins, usually damaged or misfolded (Hossain et al., 2020).

#### DNA Repair and Chromatin Remodeling

The peptide treatment could cause direct or indirect damage to fungal DNA. Indeed, at 3 h of peptide treatment, we found that 16 genes involved in DNA repair, remodeling, and maintenance of chromatin structure were differentially expressed (10 up- and 6 down regulated).

#### Cell Wall Biogenesis, Degradation and Remodeling, and Melanin Biosynthesis

The fungus reacts to peptide treatment by inducing genes involved in cell wall protection, biosynthesis, and degradation. We observed the upregulation of several genes involved in melanin biosynthesis as well as the overexpression of two CHS encoding genes. Indeed, two days after peptide treatment, we observed browning of the treated mycelia, thus confirming this fungal response (Supplementary Figure 7).

An early general remodeling/reorganization of the fungal cell wall after peptide treatment is also suggested by the observed upregulation of a gene encoding a glycosyl phosphatidylinositol anchored membrane protein, which showed to participate in fungal cell wall biosynthesis and specifically in polysaccharide remodeling (Li et al., 2018; Muszkieta et al., 2019), and by the differential regulation of several genes involved in 1,3 and 1,6 β-glucan synthesis and degradation (glucanases).

Fungal β-(1,3)-glucanases may also play key roles in the mobilization of β-glucans, in response to carbon starvation and energy source exhaustion, and immediately before fungal cell autolysis (Martin et al., 2007).

#### Fatty Acid Biosynthesis

One of the most probable effects of peptaibols on microorganisms is the damage at the membrane level. At 3 h after peptide treatment, *P. oryzae* upregulated six genes possibly involved in cell membrane lipid biosynthesis. In particular, we found as upregulated two fatty acid synthase genes and the genes encoding a fatty acid synthase S-acetyl transferase, a PKS-NRPS TAS1 synthetase, corresponding to a beta-ketoacyl synthase catalyzing the condensation of malonyl-ACP with the growing fatty acid chain and implicated in a mitochondrial pathway for fatty acid synthesis (Zhang et al., 2005), a linoleate diol synthase, polyunsaturated fatty acid of the cell membrane lipids, and a phosphatidyl-serine decarboxylase, playing a pivotal role in phospholipid synthesis of mitochondria (Schuiki and Daum, 2009).

A gene encoding the phosphatidylinositol transfer protein was also upregulated. This protein is involved in regulating sterol biosynthesis and phospholipid composition of plasma membranes (Van Den Hazel et al., 1999) and may also provide a possible mechanism for multidrug resistance altering plasma membrane composition (Van Den Hazel et al., 1999).

Besides, we also found four differentially expressed genes (two up- and two downregulated) encoding lipases, triacylglycerol acyl hydrolases involved in the hydrolysis of fats and oils to produce glycerol and free fatty acids (Singh and Mukhopadhyay, 2012).

#### Proteases and Amino Acid Transporters

The peptide treatment strongly affected the expression of many protease encoding genes. In particular, we observed the up- and down-regulation of 21 and 10 genes, respectively. The treatment also strongly affected the expression of genes encoding amino acid permeases and transporters, with four up- and eight downregulated genes.

#### Ion Efflux Transporters

Twelve genes encoding ion efflux transporters have been found to be upregulated after peptide treatment, six of them being calcium (Ca^2+^) transporters involved in maintaining Ca^2+^ homeostasis for growth, virulence and stress responses of fungi (Liu et al., 2015). Calcium acts as a second messenger in fungi, playing an essential role in cell survival also in response to stress induced by ROS. Besides, calcium and some fungal calcium signaling pathway components mediate fungal resistance to antifungal drugs (Liu et al., 2015).

### Quantitative PCR Validation of the RNA-seq Results

To validate the RNA-seq results, we analyzed the relative expression of 11 selected genes by quantitative PCR. As expected, eight genes resulted upregulated by the Pep 4Rink treatment, four of them with a relative expression higher than 10, while three genes resulted downregulated by the treatment, although one of them with a relative expression superior to 0.5 (Supplementary Figure 8).

### Ultrastructure of *Pyricularia oryzae* Conidia and Hyphae Treated With Pep 4Rink

Transmission electron microscopy observations of fungal conidia and hyphae exposed to the Pep treatment allowed us to evidence various ultrastructural modifications reported as cytological markers of autophagy in fungi (Pollack et al., 2009).

**FIGURE 8:**
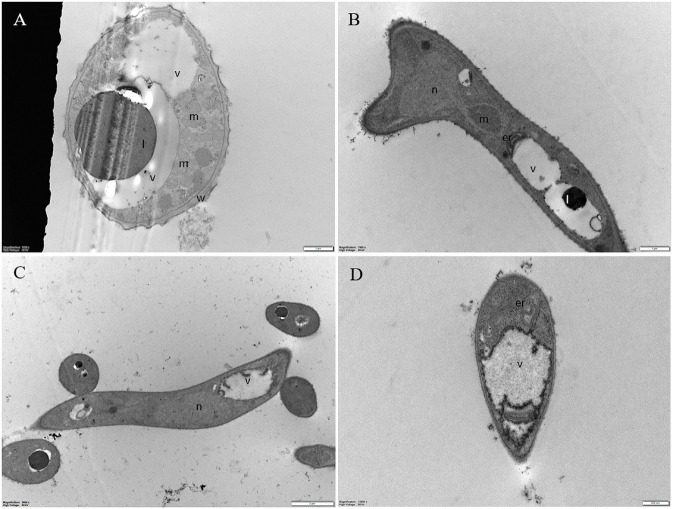
Representative TEM micrographs of *Pyricularia oryzae* untreated conidia and hyphae. **(A)** Conidia are featured with regular submicroscopic structure and well-identifiable organelles. The numerous mitochondria with membranous cristae indicate intense cell metabolism. **(B-D)** Hyphae, at 24 **(B)**, 36 **(C)** and 48 **(D)** hours after the beginning of conidial germination, show electron dense cytoplasm and well defined vacuoles. Er: endoplasmic reticulum; l: lipid drop; m: mitochondrion; n: nucleus; v: vacuole. In A and B, magnification bars correspond to 1μm; in C and D magnification bars correspond, respectively, to 2 μm and 500 nm.

Untreated conidia and hyphae displayed regular submicroscopic structure (Figure 8). The conidial wall and plasma membrane appeared intact and organelles were identifiable in the cytoplasm (Figure 8A). Hyphae were surrounded by a melanized cell wall and showed electron-dense cytoplasm (Figures 8B-D) with well-preserved organelles and vacuoles (Figures 8B-D).

**FIGURE 9:**
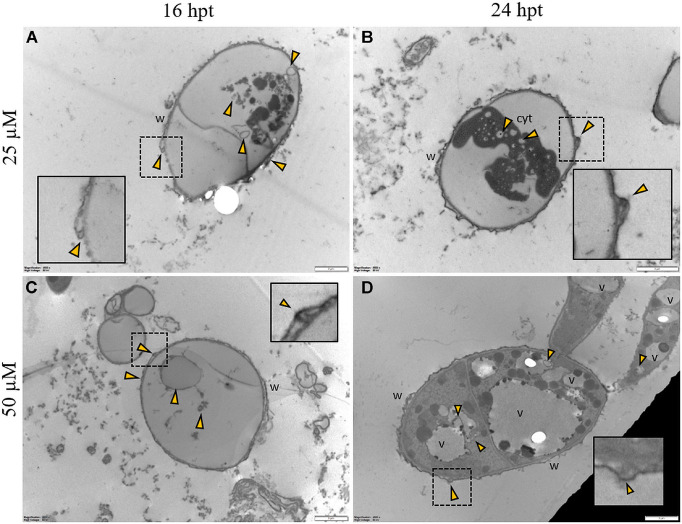
Representative TEM micrographs of *Pyricularia oryzae* conidia treated with Pep 4Rink 25 μM **(A,B)** or 50 μM **(C,D)** and observed at 16 or 24 h post treatment (hpt). Regardless of the different treatments, conidia appear empty **(A,C)** or contain condensed cytoplasm **(B)** and vesicles (**A-D**, yellow arrowheads). In panel **(D)** a germinating conidium presents abnormal vacuolation. Small vesicles (**A-D**, yellow arrowheads) are observed between the two cell wall layers. In insets, areas of interest of panels **(A-D)** are magnified. Cyt: cytoplasm; v: vacuole, w: wall. Magnification bars correspond to 2 μm.

**FIGURE 10:**
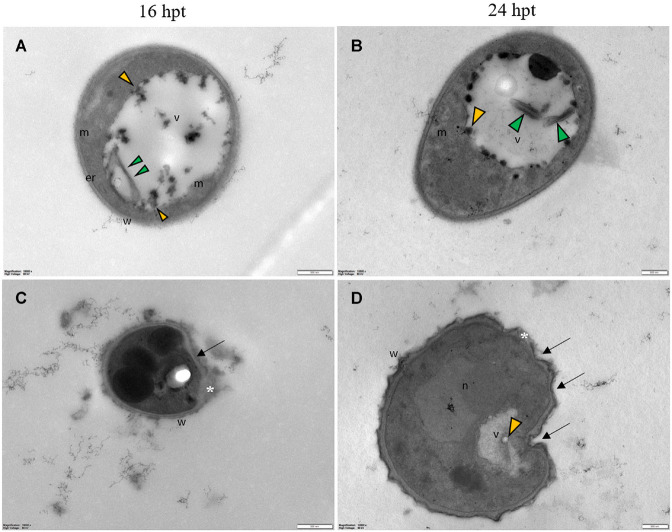
Representative TEM micrographs of *Pyricularia oryzae* hyphae treated with Pep 4Rink 25 μM and observed at 16 **(A,C)** or 24 hpt **(B,D)**. Hyphae show vacuoles containing membranous structures (**A–D**, green arrowheads) and vesicles (**A–D**, yellow arrowheads); the wall is distorted (arrows) and disrupted in some points (**C,D**, asterisk). Cyt: cytoplasm; er: endoplasmic reticulum; m: mitochondrion; n: nucleus; v: vacuole, w: wall. Magnification bars correspond to 500 nm.

**FIGURE 11:**
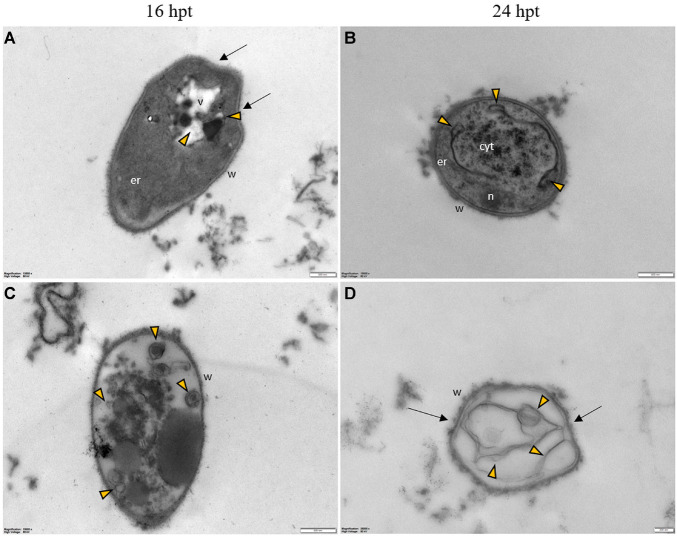
Representative TEM micrographs of *Pyricularia oryzae* hyphae treated with Pep 4Rink 50 μM and observed at 16 **(A,C)** or 24 hpt **(B,D)**. Hyphae exhibit significant ultrastructural changes, similar to what described in samples treated with Pep 4Rink 25 μM, such as distortion of the cell wall (**A,D**, arrows) and abnormal vacuolation (**A-D**, yellow arrowheads).

Regardless of the Pep concentration (25 or 50 μM) and the duration of the treatment (16 or 24 h), TEM observations revealed severe damages to *P. oryzae* conidia (Figure 9) and hyphal cells (Figures 10, 11). The conidial wall appeared severely modified, as the outer layer was detached from the inner layer, and small vesicles were observed between (Figures 9A-D). Conidia often had dense cytoplasm surrounding microvesicle-like bodies (Figures 9A,B) or appeared empty (Figure 9C). Otherwise, conidia contained membranous vesicles (Figures 9A,C) or multiple vacuoles (Figure 9D). Hyphae often displayed an apparent well-preserved morphology (Figures 10A,B); nevertheless, membranous autophagic structures and vesicles were visible inside the vacuoles (Figures 10A,B). In other hyphal cells, the wall was ruined (Figures 10C,D) or distorted (Figures 10C,D, 11A,D). Polymorphic vesicles (Figure 11C), membranous autophagic structures (Figure 11B), and multi-lamellar bodies (Figure 11D) were often found in the cytoplasm.

## Discussion

*Trichoderma* spp. antibiotic molecules called peptaibols were evaluated as effective fungicides to protect rice plants from infection by an important fungal pathogen like *P. oryzae*. We tested both *in vitro* and *in vivo* several analogs of the *T. longibrachiatum* peptaibol trichogin, designed by substituting specific residues. Almost all peptides able to strongly inhibit fungal growth have a Lys residue in positions 2 and/or 6. Peptides with a Lys in position 5 (Pep 3 and Pep 6) were only partially effective, except when in combination with a Lys in position 2 (Pep 5) or 6 (Pep 4 and modified analogs), thus suggesting that residues 2 and 6 are crucial for the antimicrobial activity of the trichogin analogs (Table 1). This conclusion partially agrees with observations performed on *Botrytis cinerea* treated with some of the peptides used in this work. In this case, the Lys residue at position 6 was considered crucial to increase peptide efficacy (De Zotti et al., 2020).

Light microscopy analyses performed on *P. oryzae* conidia treated with the effective peptides highlighted that treated spores could not germinate and presented morphological changes such as densely agglutinated cytoplasm, separated from the cell wall. Treated spores were also auto-fluorescent, thus indicating their non-viability. Conformational analysis of the peptides in the presence of the fungus, performed by circular dichroism, showed that the effective peptides retained their well-developed helical conformation, which appears therefore essential for their antifungal activity.

Most peptides found to inhibit fungal growth *in vitro* were also effective *in planta*. In the plant microenvironment, several factors can affect the interaction between peptides and pathogens. Peptide degradation by proteases, subtraction by waxes, or tissue absorption might occur, thus decreasing the effective concentration of the active compound (Zeitler et al., 2013). Interestingly, eight peptides found to reduce *P. oryzae* disease symptoms on detached barley leaves significantly were also able to reduce blast symptoms on leaves of rice seedlings, but to a different extent. Epicuticular waxes are known to influence leaf wettability strongly. Wetting of the hydrophobic leaf cuticle is usually minimal (Qin et al., 2011). As expected, when we treated barley leaves with the peptide solution, the water drop tended to cluster together, forming a spherical water bead. However, after peptide treatment of rice leaves, we observed a partial diffusion of the drop on the leaf surface, which appeared more uniformly wetted (not shown). These observations suggest that the peptide solution interacts with a different tension with the barley and rice leaf surface. Thus, the effectiveness of the treatment could be at least in part affected by the uneven distribution of the peptide. Similarly, the contact surface of fungal conidia suspension is larger in rice than barley, possibly favoring the infection process. However, it should be considered that our experiments were performed on detached leaves or seedlings. Further tests on plants under controlled environment or field conditions are needed to confirm the effectiveness of our peptides.

Many studies on AMPs focused on alterations of the plasma membrane. This paper undertook a thoroughly transcriptomic analysis on *P. oryzae* mycelia treated with Pep 4Rink to better understand the peptides’ mode of action. Due to their cationic, amphipathic and hydrophobic properties, AMPs would interact with the negatively charged phosphate groups of the microbial cell membrane, causing membrane damage and cell lysis (Muñoz et al., 2013; Wang et al., 2017; Avci et al., 2018). Our transcriptomic analysis measured an upregulation of genes involved in cell membrane lipid biosynthesis (such as fatty acid synthases) and the GO term “membrane” was also enriched in upregulated genes. This result suggests that our peptide could indirectly affect membrane integrity. It is widely reported that fatty acids, depending on their properties (i.e., chain length, degree, position and orientation of unsaturation) as well as those of their target, are able to interact with cell membranes (Bhattacharyya et al., 2020), provoking structural perturbation and the consequent loss of functionality (Liu et al., 2013; Bae and Rhee, 2019). The upregulation of genes encoding fatty acid synthases in treated *P. oryzae* cells correlates with membrane rearrangement of to form vesicles and autophagic bodies (Polyansky et al., 2020), observed through the TEM analysis.

Our transcriptomic analysis did not highlight any effect on the expression of genes involved in the biosynthesis of ergosterol, one of the main components of the cell membrane (Jordá and Puig, 2020), which is unexpected considering previous literature findings. For example, Wang et al. (2017) observed a downregulation of ERG genes in *C. albicans* treated with the cationic peptide MAF-1A. Thus, we can hypothesize that ergosterol is not the main target of our peptides, as previously assumed in the case of another family of membrane-active synthetic ultrashort cationic lipopeptides (Makovitzki et al., 2007).

Although AMPs have been mainly recognized by their ability to interact with cell membranes disrupting their integrity, some AMPs can enter into microbial cells by pore-forming or non-pore-forming mechanisms (Avci et al., 2018), thus interacting with intracellular targets and inducing cell injuries and eventually cell death (Muñoz et al., 2013; Avci et al., 2018). The two major routes for AMPs into the cells are endocytosis or direct cell membrane penetration, favored by high peptide concentrations (Muñoz et al., 2013; Avci et al., 2018). Our fluorescence microscopic observations indicate that the peptide analogs can localize at the cell wall of *P. oryzae* conidia, likely attracted by the cell wall negative charge, and intracellularly in the agglutinated cytoplasm. Interestingly, TEM observations performed on treated *P. oryzae* conidia revealed ultrastructural modifications at the cell wall and cytoplasm levels. In particular, the small vesicles observed at the conidial cell wall could be consistent with a possible AMP uptake toward the cytoplasm by vesicular transport (Rodrigues et al., 2008; Rodrigues and Casadevall, 2018).

A similar intracellular localization was also observed with other cationic peptides such as PAF26 in *Neurospora crassa* (Muñoz et al., 2013). Following the model described by these authors, sub-inhibitory concentrations of PAF26 cause the depolarization of the plasma membrane, which allows the endocytic internalization of the peptide, a step associated with the increasing cytoplasmic concentration of calcium ions. PAF26 would initially accumulate in expanding vacuoles, and its subsequent transport in the cytoplasm permeates the intracellular membranes (Muñoz et al., 2013). Our transcriptomic analysis seems to follow this model since we also observed already at 3 h the up-regulation of genes involved in Ca^2+^ transport, vesicular formation, and transport and endocytosis.

Interestingly, it has been proposed that peptaibols, cyclic lipopeptides and other AMPs can cause cell death in addition to their membrane disruption effect (Wang et al., 2017). For example, several biochemical hallmarks of PCD or metacaspase-independent apoptosis have been identified in plant pathogens such as *Botrytis cinerea* when treated with trichokonins, peptaibols produced by *T. pseudokoningii* (Shi et al., 2012; Zhao et al., 2018). The cyclic lipopeptide fengycin from *Bacillus subtilis* induces ROS bursts, membrane damage, chromatin condensation, and cell death in *M. oryzae* hyphae (Zhang and Sun, 2018). Besides, the active transfer of the PAF26 peptide in *N. crassa* cytosol has been demonstrated to coincide with cell death (Muñoz et al., 2013). ROS formation, such as hydrogen peroxide and hydroxyl radicals, can be directly or indirectly associated with cell death (Shi et al., 2012; Gonçalves et al., 2017). Indeed, ROS are known to elicit damage to cell membranes and mitochondria, proteins, lipids and nucleic acids, resulting in irreversible cell damage and loss of viability (Redza-Dutordoir and Averill-Bates, 2016; Wang et al., 2017). In our transcriptomic analysis, we observed the upregulation of SOD, AOX, galactose oxidase and Cu radical oxidase encoding genes and the downregulation of CAT and GPX, thus suggesting a rapid burst of H_2_O_2_ concentration in the treated mycelium.

A well-characterized response of eukaryotic organisms to ROS accumulation is the rapid induction of oxidative stress, detoxification and repair proteins. In our transcriptomic analysis, we observed the upregulation of several genes dealing with the synthesis and degradation of glutathione, a key molecule in the detoxification of toxic compounds and response to oxidative stress. Besides, we found many genes involved in DNA repair, remodeling, and maintenance of chromatin structure as differentially expressed, thus suggesting that DNA damage was likely occurring in fungal cells, probably contributing to cell death. Whether the positively charged peptide directly causes DNA damage or the damage is rather an indirect effect of ROS accumulation, which at high levels is lethal for fungal cells (Roberto et al., 2020) needs to be further investigated.

We can also speculate considering the induction of fungal cell death due to the up-regulation of several autophagy-inducing genes and genes encoding for proteasome regulatory subunits and components. In eukaryotic cells, ubiquitin-26S proteasome system (UPS) and autophagy are mostly responsible for protein turnover (Wang and Schippers, 2019). Proteins targeted for degradation by the UPS are labeled with ubiquitin to enable recognition by the proteasome, which usually degrades damaged and misfolded proteins (Hossain et al., 2020). Autophagy is a conserved recycling process triggered by stress, particularly nitrogen starvation, to support cell survival (Zhu et al., 2018). Autophagy can recycle cytoplasmic material such as larger protein complexes and insoluble aggregates, abnormal proteins, other macromolecules and entire dysfunctional organelles such as mitochondria and peroxisomes (Marshall and Vierstra, 2019) that could be directly or indirectly damaged by the peptide treatment. However, although the central physiological role of autophagy is the recycling of amino acids from proteins for survival during nitrogen starvation, this mechanism also plays an important role in the form of non-apoptotic PCD, which is a cell survival strategy widely occurring in fungal conidia (Khan et al., 2012). Indeed, autophagy has also been called type II PCD or autophagic cell death (Pollack et al., 2009) and is required in *P. oryzae* for conidia PCD, differentiation of a functional appressorium, and thus for successful rice infection (Veneault-Fourrey et al., 2006; Kershaw and Talbot, 2009). The autophagic PCD hypothesis is supported by the enrichment in our FunCat and GO analyses of upregulated categories such as calcium ion transport and vesicle formation and transport, which could be involved in the activation of autophagic-like cell death in fungi (Shi et al., 2012) and autophagosome formation, respectively. To further support this hypothesis, ultrastructural investigations performed on treated conidia and hyphae revealed the presence of various vesicular and multi-lamellar bodies considered as autophagic markers in fungi (Pollack et al., 2009). Similar to those described in our work, cytological abnormalities were reported in conidia and hyphae of *M. oryzae* and *Fusarium graminearum* during the interaction with antagonistic microorganisms, respectively *B. subtilis* (Sha et al., 2016) and *Streptomyces hygroscopicus* (Wang et al., 2021). Such morphological modifications were associated with autophagy processes following the secretion of antibiotic compounds by the antagonists.

More than 40 autophagy-related (ATG) genes have been identified in fungi (Roberto et al., 2020). Our transcriptomic analysis found that ATG3, ATG4, ATG7, ATG9 and ATG17 encoding genes were upregulated. In particular, ATG4 encodes a cysteine protease necessary for processing ATG8, an essential gene for autophagosome synthesis and thus for autophagy pathway activation (Liu et al., 2010; Ren et al., 2018; Roberto et al., 2020). Besides, in *P. oryzae*, ATG3 and ATG7 putatively play a role in phagophore and autophagosome expansion, ATG9 in recycling, and ATG17 in the initiation of autophagy (Kershaw and Talbot, 2009). Interestingly, autophagy is also induced during cell death by heterokaryon incompatibility in the fungus *Podospora anserina* (Pinan-Lucarré et al., 2003). Accordingly, our transcriptomic analysis found that seven genes encoding heterokaryon incompatibility (HET) proteins are upregulated. The overexpression of HET encoding genes has been shown to induce cell death by incompatibility hallmarks such as autophagy and increased vacuolization (Paoletti and Clavé, 2007). Indeed, PCD plays a major role in HET. During this reaction, the induction of PCD is very rapid, and apoptosis-associated morphological changes such as cytoplasm condensation and shrinkage of the plasma membrane occur (Shlezinger et al., 2012; Gonçalves et al., 2017). These phenotypes were also observed in *P. oryzae* spores treated with our peptide analogs.

In our RNA-seq analysis, we observed that many genes related to cell wall biosynthesis and remodeling were differentially expressed after exposure to Pep 4Rink. In particular, we found two chitin synthase (CHS) genes as upregulated, namely MGG_01802, corresponding to CHS1, and MGG_09551, corresponding to CHS3 (Kong et al., 2012). CHS1 and CHS3 encoding genes have similar expression patterns in *P. oryzae*, and the chs1/chs3 double mutant showed increased sensitivity to hyperosmotic and oxidative stresses, indicating that both genes may play a role in cell wall modification and reinforcement in response to stresses (Kong et al., 2012).

Besides, several genes involved in 1,3 and 1,6 β-glucan synthesis and degradation (glucanases) were differentially regulated. Indeed, β-glucans are major components of fungal cell walls and cell wall growth, and extension represents a delicate balance between the hydrolysis of the existing cell wall and new wall synthesis (Martin et al., 2007). In particular, glucan sheath apposition might protect the fungus from external stressors (Martin et al., 2007).

Similar results to those obtained in our transcriptomic analysis have been obtained in *Candida albicans* and *Penicillium digitatum* treated with AMPs, namely upregulation of β-glucan synthase genes and induction of several CHS genes, respectively (Muñoz et al., 2013; Wang et al., 2017). On the whole, the fungal cell wall appears as an essential and dynamic structure. Fungi constantly remodel it by breaking and reforming polysaccharide chemical bonds to maintain its structural integrity. The fungal cell wall can be considered a general defense barrier to counteract AMP action, and its strengthening is a common response after exposure to AMPs (López-García et al., 2010), likely constituting a protective shield against toxic peptides. In this regard, the strong upregulation of several melanin biosynthesis and laccase genes was observed in our RNA-seq analysis, a result confirmed by the browning of the treated mycelium observed two days after peptide treatment. Melanin in fungi is considered to play a protective role (Cordero and Casadevall, 2017; Cui et al., 2020) and strengthen the cell wall by preventing the penetration of the peptide into the fungal cells. Fungal laccases are also reported to perform several functions such as pigmentation (dihydroxynaphthalene melanin, produced against environmental stress) and detoxification (Lu et al., 2017).

Among the detoxification strategies activated by fungi, ABC transporters could play a significant role. Indeed, both FunCat and manual analysis of DEGs highlighted that several *P. oryzae* genes encoding ABC-transporters were upregulated by pep 4Rink treatment.

The protective mechanisms described above, i.e., cell wall reinforcement, melanin apposition and activation of ABC transporters, could be differentially activated in the fungal strains and could confer susceptibility or tolerance to different peptides, as observed in our *in vitro* experiment. However, further studies are needed in order to clarify this particular aspect.

Taken together, our results support the hypothesis that our peptide analogs cause severe oxidative stress in the fungus, with possible direct or indirect damage to the cell wall, plasma membrane, DNA and other macromolecules. Besides, possibly accumulating in the fungal cells up to a critical concentration (Tavano et al., 2015), the peptide analogs rapidly trigger autophagic cell death. The fungus reacts to the toxic molecule by strengthening and/or remodeling its cell wall, mainly by depositing melanin and activating transporters and detoxification mechanisms.

Nevertheless, a significant number (about 35%) of the genes identified in our transcriptomic analysis as differentially expressed have unknown functions; therefore, the peptide treatment could induce other not yet disclosed effects.

In conclusion, we have identified several peptides able to significantly reduce rice blast symptoms that seem promising molecules for the future development of effective bio-pesticides against *P. oryzae* under open-field conditions.

## Data Availability Statement

The RNA-seq datasets presented in this study can be found in an online repository. The name of the repository and accession number can be found below: ArrayExpress database at EMBL-EBI (www.ebi.ac.uk/arrayexpress), E-MTAB-10927.

## Author Contributions

LS, FF, and MD did the conceptualization. LS, RG, RC, AQ, RM, and MD performed the methodology and investigated the data. LS, RG, RC, AQ, RM, MD, and FF carried out the data curation. LS, RG, RM, and MD wrote the original draft. LS, FF, MD, RM, RC, AQ, ST, HN, and VV wrote, reviewed, and edited the manuscript. LS supervised the data. LS and HN carried out the project administration and funding acquisition. All authors have read and agreed to the published version of the manuscript.

## Conflict of Interest

The authors declare that the research was conducted in the absence of any commercial or financial relationships that could be construed as a potential conflict of interest.

## Publisher’s Note

All claims expressed in this article are solely those of the authors and do not necessarily represent those of their affiliated organizations, or those of the publisher, the editors and the reviewers. Any product that may be evaluated in this article, or claim that may be made by its manufacturer, is not guaranteed or endorsed by the publisher.

## Footnotes

1
https://www.ebi.ac.uk/Tools/hmmer/search/hmmscan

2
https://elbe.hki-jena.de/fungifun/

## Supplementary Material

Supplementary Figure 1 Disease Severity Index caused by *Pyricularia oryzae* on rice leaves. DS0: no symptoms; DS1: brown spots; DS2: small necrotic lesion with yellow margins; DS3: necrotic lesion with gray center and yellow edge; DS4: extended score 3 lesions - great part of leaf blade/surface yellowed and necrotized (from Pak et al., 2017).

Supplementary Figure 2 Representative lesions caused by *Pyricularia oryzae* (IT10 strain) on barley leaves (cv. Alora) untreated (Control) or treated with 50 μM peptides 7 days after inoculation. Almost no lesions were observed with Pep 2Rink and Pep 4Rink.

Supplementary Figure 3 Representative lesions caused by *Pyricularia oryzae* (IT10 strain) on rice leaves (cv. Vialone Nano cultivar) untreated (Control) or treated with 50 μM peptides at 8 days post inoculation. Compared to Control and Pep 3, used as negative control, reduced lesions were observed with Pep 2Rink, 4C2 and 5.

Supplementary Figure 4 Optical microscopy (Leica; Leica Microsystems) pictures representing *Pyricularia oryzae* (IT10 strain) conidia after 48 h of incubation with 50 μM of each peptide in Potato Dextrose Broth (PDB) medium. **(A)** untreated control; **(B)** Pep 2; **(C)** Pep 4; **(D)** Pep 4Rink; **(E)** Pep 4C; **(F)** Pep 5; **(G)** Pep 6; **(H)** Pep 8ApiC; **(I)** Pep 19; **(J)** Pep 22Rink.

Supplementary Figure 5 Optical **(A-C)** and fluorescence **(D-F)** microscopy pictures of *P. oryzae* (IT10 strain) spores untreated or treated with Pep 4Rink after 48 h of incubation in Potato Dextrose Broth (PDB) medium. Untreated *P. oryzae* spore germinated and not showing auto-fluorescence (A-D), hence indicating its viability. When treated with Pep 4Rink, the *P. oryzae* spore showed cytoplasmic agglutination under optical microscope **(B)** and auto-fluorescence under fluorescent microscopy **(E)**, thus indicating its non-viability. Fluorescent microscopy observation of a *P. oryzae* spore treated for 48 h with Pep 4Rink conjugated with the FITC fluorophore showed that the peptide was able to permeabilize the cell membrane, being located intracellularly in the agglutinated cytoplasm, and was also located at the spore cell wall **(F)**.

Supplementary Figure 6 Principal component analysis (PCA) of *P. oryzae* control (C) and Pep 4Rink treated (T) biological replicates at 3 h after treatment.

Supplementary Figure 7 *Pyricularia oryzae* mycelium untreated **(A-C)** or treated with 50 μM of Pep 4Rink **(B-D)** after 6 h **(A,B)** or 48 h **(C,D)** of growth in complete medium (CM).

Supplementary Figure 8 RT-qPCR analysis of some selected *Pyricularia oryzae* genes performed on a Rotor-Gene Q 2plex (Qiagen) by using as template RNA extracted from 3 days old mycelium of *P. oryzae* untreated or treated for 3 h with pep 4Rink. **(A)** Relative expression of selected genes found as upregulated in the RNA-seq analysis; **(B)** Relative expression of selected genes found as downregulated in the RNA-seq analysis. Relative expression was analyzed using the Rotor-Gene v. 2.0.3.2 software (Qiagen) and the tool REST by using *P. oryzae* actin (MGG_03982) and glyceraldehyde-3-phosphate dehydrogenase (MGG_01084) as normalizers. Error bars indicate standard errors calculated from two biological replicates.

Supplementary Table 1 List of primers used in the RT-qPCR analysis.

Supplementary Table 2 List of all the differentially expressed genes (DEGs) identified by RNA seq analysis at 3 h after *Pyricularia oryzae* treatment with Pep 4Rink.

Supplementary Table 3 FunCat Categories assigned to up-regulated genes at 3 h post treatment. Categories were ranked based on the adjusted *p*-value <0.05 calculated with the Fisher’s enrichment exact test. The percentage of genes assigned to a specific category was calculated with respect to the total number of genes assigned to all the categories identified.

Supplementary Table 4 FunCat Categories assigned to down-regulated genes at 3 h post treatment. Categories were ranked based on the adjusted *p*-value <0.05 calculated with the Fisher’s enrichment exact test. The percentage of genes assigned to a specific category was calculated with respect to the total number of genes assigned to all the categories identified.

Supplementary Table 5 Go Categories assigned to up-regulated genes at 3 h post treatment. GO names were ranked based on the adjusted *p*-value <0.05 calculated with the Fisher’s enrichment exact test. The percentage of genes assigned to a specific GO name was calculated with respect to the total number of genes assigned to all the categories identified. BP = Biological Process; CC = Cellular Component; MF = Molecular function.

Supplementary Table 6 Go Categories assigned to down-regulated genes at 3 h post treatment. GO names were ranked based on the adjusted *p*-value <0.05 calculated with the Fisher’s enrichment exact test. The percentage of genes assigned to a specific GO name was calculated with respect to the total number of genes assigned to all the categories identified. BP = Biological Process; CC = Cellular Component; MF = Molecular function.

Supplementary Table 7 *Pyricularia oryzae* genes up-regulated after 3 h from peptide treatment.

Supplementary Table 8 *Pyricularia oryzae* genes down-regulated after 3 h from peptide treatment.
