# Supplementary figures and images for: Transcriptomic and Ultrastructural Analyses of Pyricularia Oryzae Treated With Fungicidal Peptaibol Analogs of Trichoderma Trichogin
Source: Front Microbiol. 2021 Oct 14;12:753202. doi: 10.3389/fmicb.2021.753202 (PMC8551967; doi:10.3389/fmicb.2021.753202)

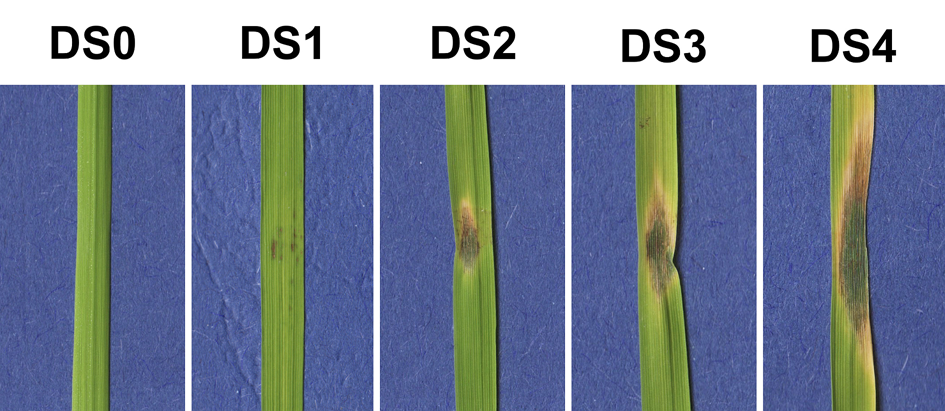

Supplement: Supplementary Figure 1 — Disease Severity Index caused by Pyricularia oryzae on rice leaves. DS0: no symptoms; DS1: brown spots; DS2: small necrotic lesion with yellow margins; DS3: necrotic lesion with gray center and yellow edge; DS4: extended score 3 lesions - great part of leaf blade/surface yellowed and necrotized (from Pak et al., 2017). [file Image_1.TIF]

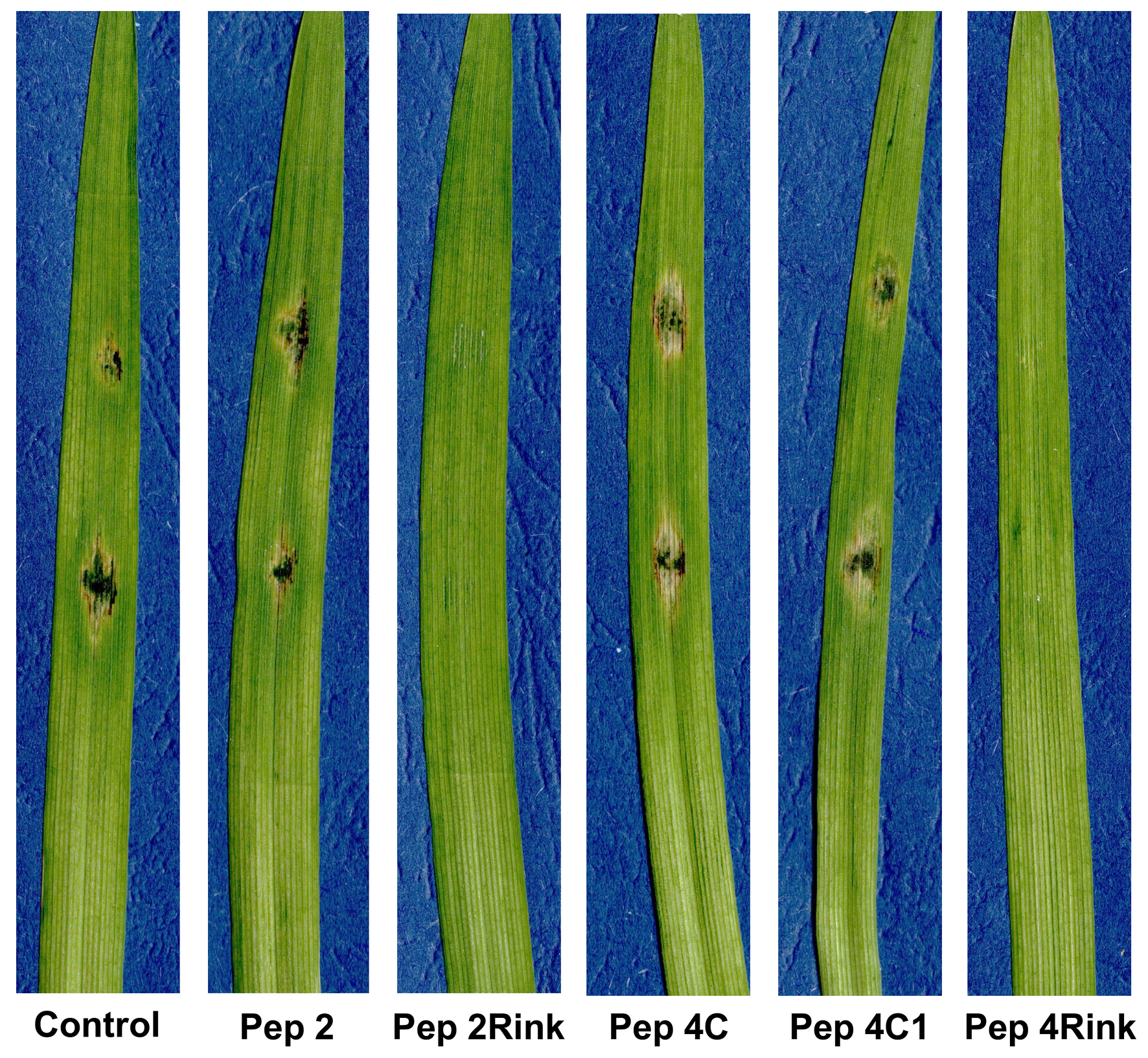

Supplement: Supplementary Figure 2 — Representative lesions caused by Pyricularia oryzae (IT10 strain) on barley leaves (cv. Alora) untreated (Control) or treated with 50 μM peptides 7 days after inoculation. Almost no lesions were observed with Pep 2Rink and Pep 4Rink. [file Image_2.TIF]

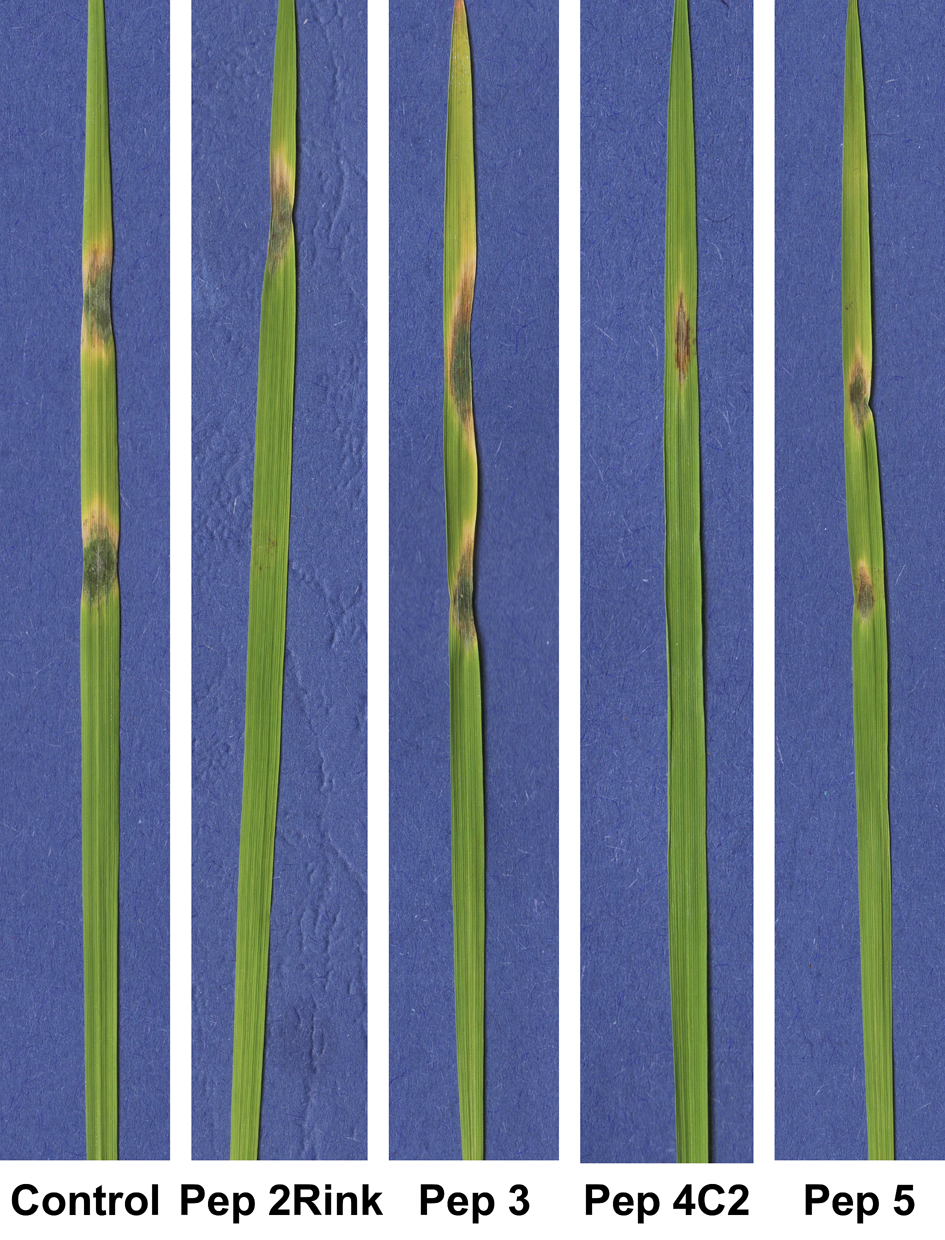

Supplement: Supplementary Figure 3 — Representative lesions caused by Pyricularia oryzae (IT10 strain) on rice leaves (cv. Vialone Nano cultivar) untreated (Control) or treated with 50 μM peptides at 8 days post inoculation. Compared to Control and Pep 3, used as negative control, reduced lesions were observed with Pep 2Rink, 4C2 and 5. [file Image_3.TIF]

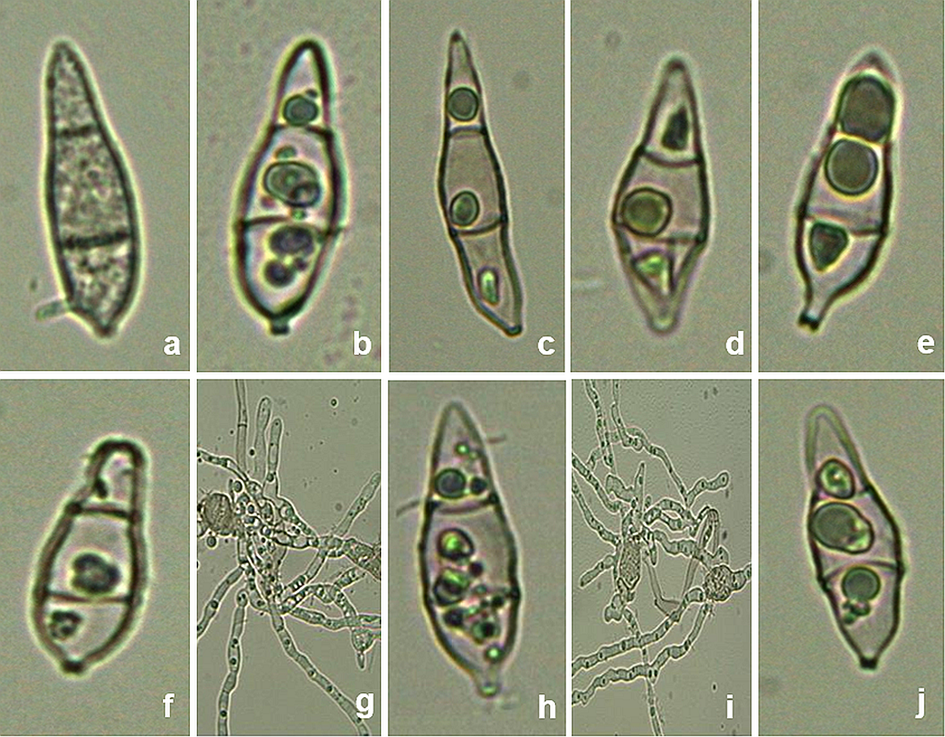

Supplement: Supplementary Figure 4 — Optical microscopy (Leica; Leica Microsystems) pictures representing Pyricularia oryzae (IT10 strain) conidia after 48 h of incubation with 50 μM of each peptide in Potato Dextrose Broth (PDB) medium. (A) untreated control; (B) Pep 2; (C) Pep 4; (D) Pep 4Rink; (E) Pep 4C; (F) Pep 5; (G) Pep 6; (H) Pep 8ApiC; (I) Pep 19; (J) Pep 22Rink. [file Image_4.TIF]

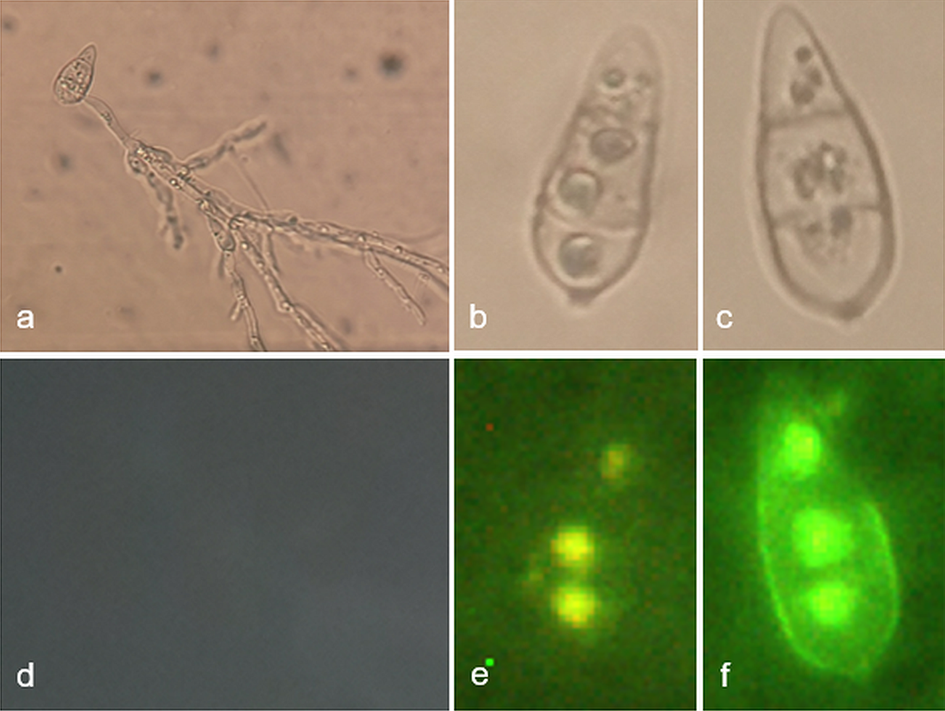

Supplement: Supplementary Figure 5 — Optical (A-C) and fluorescence (D-F) microscopy pictures of P. oryzae (IT10 strain) spores untreated or treated with Pep 4Rink after 48 h of incubation in Potato Dextrose Broth (PDB) medium. Untreated P. oryzae spore germinated and not showing auto-fluorescence (A-D), hence indicating its viability. When treated with Pep 4Rink, the P. oryzae spore showed cytoplasmic agglutination under optical microscope (B) and auto-fluorescence under fluorescent microscopy (E), thus indicating its non-viability. Fluorescent microscopy observation of a P. oryzae spore treated for 48 h with Pep 4Rink conjugated with the FITC fluorophore showed that the peptide was able to permeabilize the cell membrane, being located intracellularly in the agglutinated cytoplasm, and was also located at the spore cell wall (F). [file Image_5.TIF]

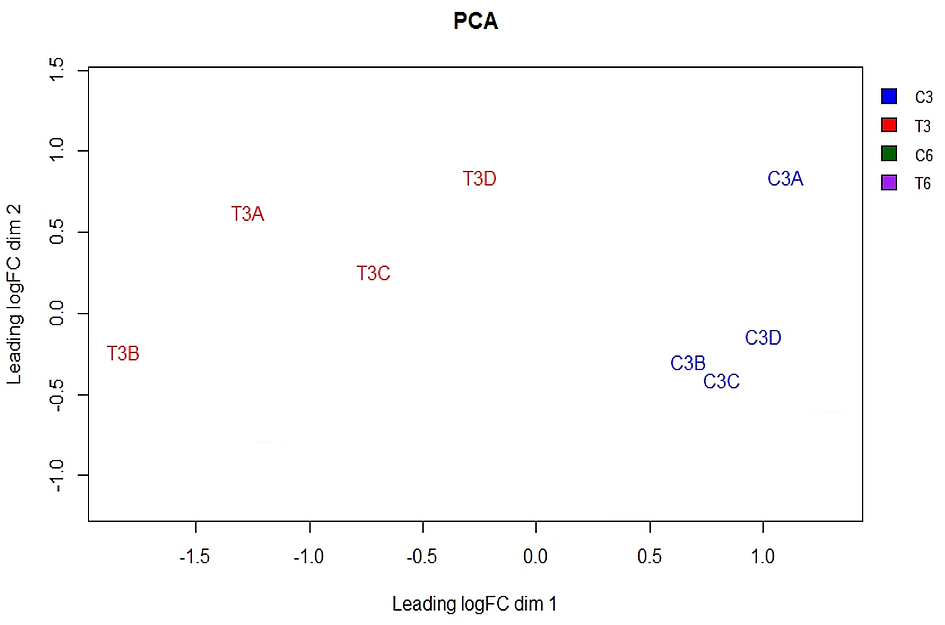

Supplement: Supplementary Figure 6 — Principal component analysis (PCA) of P. oryzae control (C) and Pep 4Rink treated (T) biological replicates at 3 h after treatment. [file Image_6.TIF]

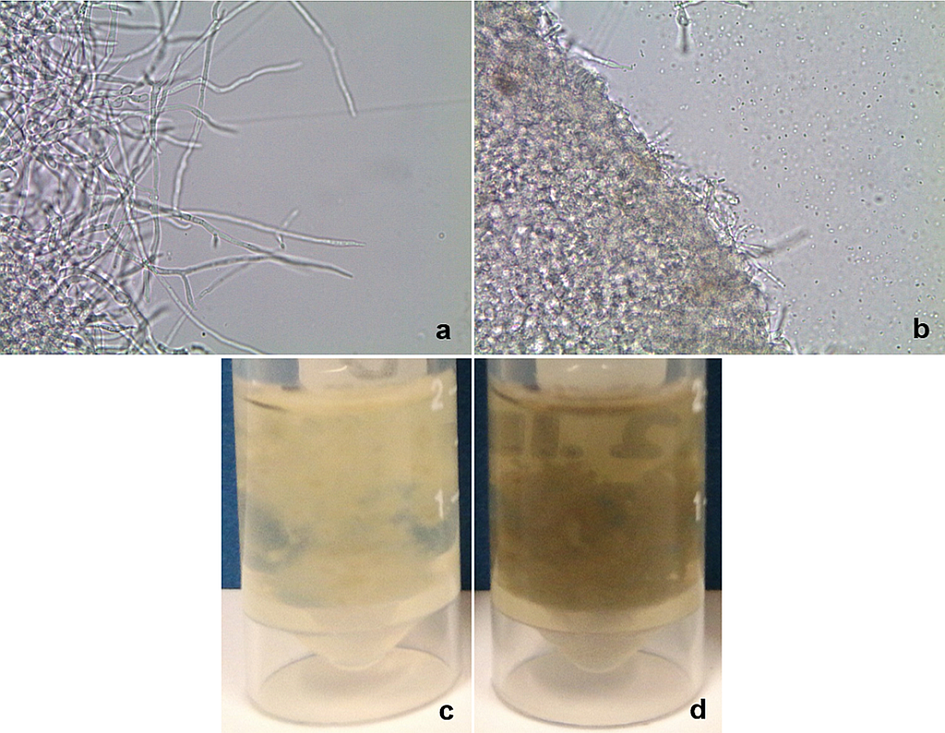

Supplement: Supplementary Figure 7 — Pyricularia oryzae mycelium untreated (A-C) or treated with 50 μM of Pep 4Rink (B-D) after 6 h (A,B) or 48 h (C,D) of growth in complete medium (CM). [file Image_7.TIF]

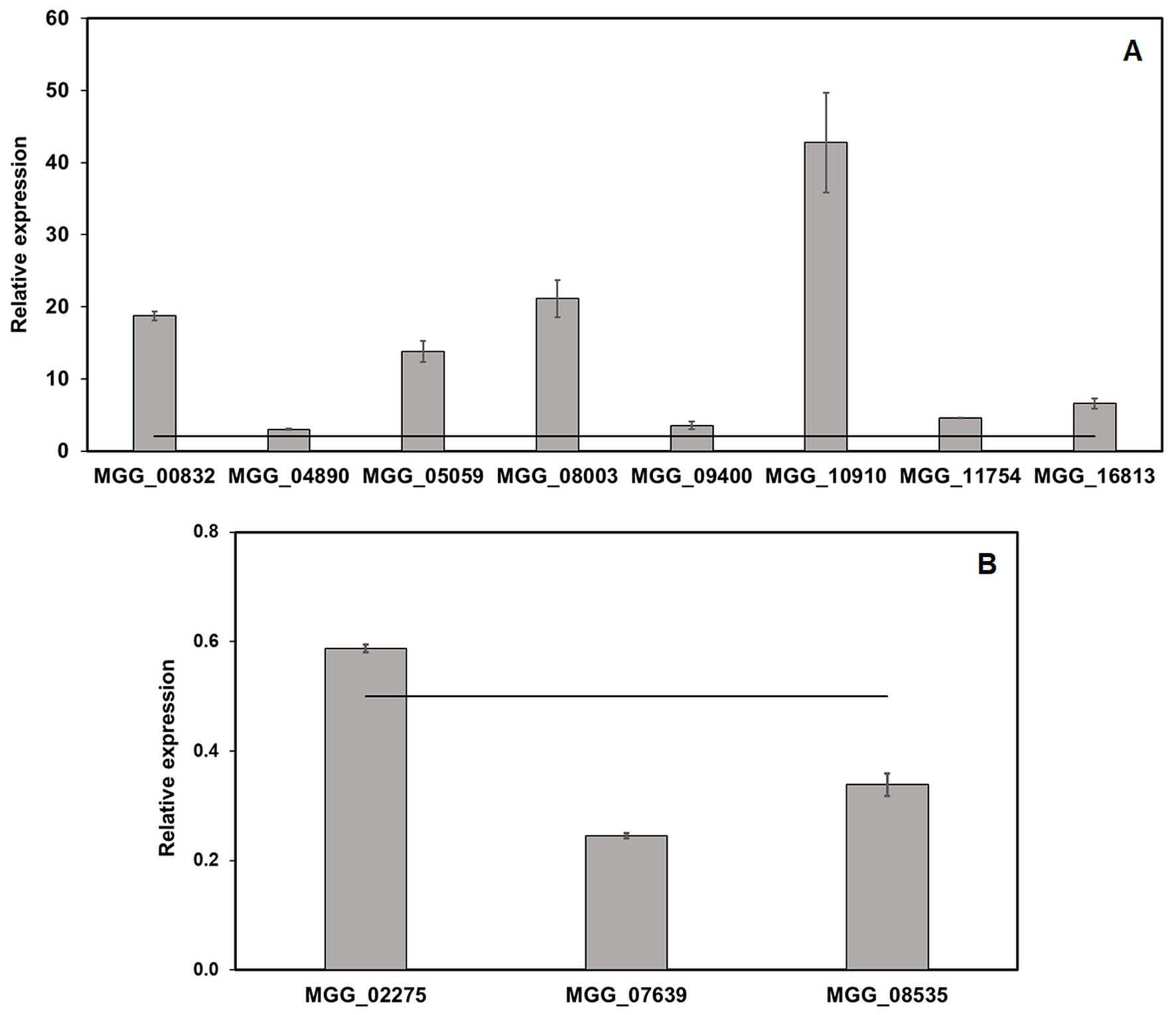

Supplement: Supplementary Figure 8 — RT-qPCR analysis of some selected Pyricularia oryzae genes performed on a Rotor-Gene Q 2plex (Qiagen) by using as template RNA extracted from 3 days old mycelium of P. oryzae untreated or treated for 3 h with pep 4Rink. (A) Relative expression of selected genes found as upregulated in the RNA-seq analysis; (B) Relative expression of selected genes found as downregulated in the RNA-seq analysis. Relative expression was analyzed using the Rotor-Gene v. 2.0.3.2 software (Qiagen) and the tool REST by using P. oryzae actin (MGG_03982) and glyceraldehyde-3-phosphate dehydrogenase (MGG_01084) as normalizers. Error bars indicate standard errors calculated from two biological replicates. [file Image_8.TIF]
