# Supplementary material for: Transcriptomic and Ultrastructural Analyses of Pyricularia Oryzae Treated With Fungicidal Peptaibol Analogs of Trichoderma Trichogin
Source: Front Microbiol. 2021 Oct 14;12:753202. doi: 10.3389/fmicb.2021.753202 (PMC8551967; doi:10.3389/fmicb.2021.753202)
Supplement: Supplementary Table 1 — List of primers used in the RT-qPCR analysis. [file Table_1.DOCX]

**Table S1**. List of primers used in the RT-qPCR analysis.

| **Gene** | **Accession number** | **Primer sequence** | **Tm (°C)** | **Size (bp)** |
| --- | --- | --- | --- | --- |
| Actin | MGG_03982 | CACCTTCTCTACCACTGCC | 59.87 | 228 |
|  |  | GATACCACCGCTCTCAAGAC | 60.37 |  |
| Glyceraldehyde-3-P dehydrogenase | MGG_01084 | CAACATTCCCTGGTCCGAG | 60.55 | 267 |
|  |  | CTCAACGATGCCAAACTTGTC | 60.55 |  |
| Pyriculariol biosynthesis protein | MGG_10910 | GAACATCCACTCCTTTATCTTTCC | 59.64 | 282 |
|  |  | AAGACTGTGCCATCCGAG | 59.47 |  |
| Cytochrome P450 | MGG_00832 | GCATCAAGCCGAACAAGAC | 59.95 | 280 |
|  |  | CGCAAACACAAACATCTCGT | 60.16 |  |
| Scytalone dehydratase | MGG_05059 | TTGACTACCGCTCCTTCCT | 60.40 | 208 |
|  |  | GTGGTGTCCTTGTACCTCTG | 60.16 |  |
| NADP/NADPH oxreductase | MGG_16813 | ACACCCACAATACCAGCC | 59.70 | 240 |
|  |  | AGTAGTGCTTGACGAGGTG | 59.50 |  |
| ABC transporter | MGG_11754 | ACCACAAGATCACGACGG | 59.79 | 300 |
|  |  | CGACAATCTTTCCATCCTTGAC | 59.82 |  |
| Uncharacterized protein | MGG_09400 | ACAAGCGACTAATCATCGGG | 60.37 | 292 |
|  |  | GATTTCCACTCCCATCCCTC | 60.01 |  |
| Plasma membrane calcium-transporting ATPase 4 | MGG_04890 | ACAACGACAAGATTCTCATCCT | 59.94 | 266 |
|  |  | CAACTCTACACTCTTTCCCGA | 59.60 |  |
| UbiA prenyltransferase | MGG_08003 | ATATGGCTCTTTACCGAGTCTG | 59.87 | 238 |
|  |  | GCTTGTTAATCCTGTCCTCCT | 59.94 |  |
| Endoprotease | MGG_02275 | TCGCCAATCGTCATCACC | 60.26 | 209 |
|  |  | TGGATTGAGTTGTTGAGCGT | 60.38 |  |
| Excitatory Amino Acid transporter | MGG_07639 | CCGCTCATTCATCACCGA | 59.95 | 202 |
|  |  | AATCAGAAAGAAGACGCCGA | 59.87 |  |
| MARVEL domain-containing protein | MGG_08535 | AATTCTCGGCTTGGGTCTC | 59.87 | 257 |
|  |  | GCCAGAGGTAGCTGAAGAC | 59.95 |  |
