# Supplementary material for: Transcriptomic and Ultrastructural Analyses of Pyricularia Oryzae Treated With Fungicidal Peptaibol Analogs of Trichoderma Trichogin
Source: Front Microbiol. 2021 Oct 14;12:753202. doi: 10.3389/fmicb.2021.753202 (PMC8551967; doi:10.3389/fmicb.2021.753202)
Supplement: Supplementary Table 3 — FunCat Categories assigned to up-regulated genes at 3 h post treatment. Categories were ranked based on the adjusted p-value <0.05 calculated with the Fisher’s enrichment exact test. The percentage of genes assigned to a specific category was calculated with respect to the total number of genes assigned to all the categories identified. [file Table_3.DOCX]

**Table S3.** FunCat Categories assigned to up-regulated genes at 3h post treatment. Categories were ranked based on the adjusted p-value < 0.05 calculated with the Fisher’s enrichment exact test. The percentage of genes assigned to a specific category was calculated with respect to the total number of genes assigned to all the categories identified.

|  | | | | |
| --- | --- | --- | --- | --- |
| **FunCat description** | **FunCat main category** | **Adjusted p-value** | **# genes / category** | **Assigned genes %** |
| ER to Golgi transport | Cellular transport, transport facilitation and transport routes | 8.936579e-21 | 41 / 73 | 5.37 |
| vesicular transport (Golgi network, etc.) | Cellular transport, transport facilitation and transport routes | 1.138331e-10 | 53 / 192 | 6.95 |
| protein transport | Cellular transport, transport facilitation and transport routes | 2.127392e-9 | 58 / 239 | 7.60 |
| proteasomal degradation (ubiquitin/proteasomal pathway) | Protein fate (folding, modification, destination) | 8.71972E-06 | 39 / 165 | 5.11 |
| intracellular transport vesicles | Biogenesis of cellular components | 0.000458514 | 13 / 33 | 1.70 |
| protein targeting, sorting and translocation | Protein fate (folding, modification, destination) | 0.000482912 | 55 / 313 | 7.21 |
| autoproteolytic processing | Protein fate (folding, modification, destination) | 0.001460184 | 10 / 23 | 1.31 |
| vesicle formation | Cellular transport, transport facilitation and transport routes | 0.0033548 | 11 / 30 | 1.44 |
| vesicle recycling | Cellular transport, transport facilitation and transport routes | 0.006685358 | 5 / 7 | 0.66 |
| protein modification | Protein fate (folding, modification, destination) | 0.006685358 | 33 / 177 | 4.33 |
| vacuolar/lysosomal transport | Cellular transport, transport facilitation and transport routes | 0.006994318 | 29 / 149 | 3.80 |
| non-vesicular ER transport | Cellular transport, transport facilitation and transport routes | 0.008045286 | 6 / 11 | 0.79 |
| protein processing (proteolytic) | Protein fate (folding, modification, destination) | 0.008045286 | 26 / 130 | 3.41 |
| endocytosis | Cellular transport, transport facilitation and transport routes | 0.009835697 | 23 / 111 | 3.01 |
| transport ATPases | Cellular transport, transport facilitation and transport routes | 0.009878852 | 20 / 91 | 2.62 |
| aliphatic hydrocarbon catabolism | Metabolism | 0.01053952 | 4 / 5 | 0.52 |
| protein binding | Protein with binding function or cofactor requirement (structural or analytic) | 0.01122315 | 178 / 1483 | 23.33 |
| transport routes | Cellular transport, transport facilitation and transport routes | 0.01146821 | 9 / 26 | 1.18 |
| cellular export and secretion | Cellular transport, transport facilitation and transport routes | 0.01203594 | 30 / 166 | 3.93 |
| N-directed glycosylation, deglycosylation | Protein fate (folding, modification, destination) | 0.01500664 | 12 / 44 | 1.57 |
| stress response | Cell rescue, defense and virulence | 0.01865747 | 41 / 258 | 5.37 |
| calcium binding | Protein with binding function or cofactor requirement (structural or analytic) | 0.02081899 | 18 / 85 | 2.36 |
| intra Golgi transport | Cellular transport, transport facilitation and transport routes | 0.02883589 | 9 / 30 | 1.18 |
| actin cytoskeleton | Biogenesis of cellular components | 0.02998304 | 22 / 117 | 2.88 |
| lipid binding | Protein with binding function or cofactor requirement (structural or analytic) | 0.03372558 | 14 / 62 | 1.83 |
| retrograde transport | Cellular transport, transport facilitation and transport routes | 0.03868706 | 4 / 7 | 0.52 |
