# Supplementary material for: Transcriptomic and Ultrastructural Analyses of Pyricularia Oryzae Treated With Fungicidal Peptaibol Analogs of Trichoderma Trichogin
Source: Front Microbiol. 2021 Oct 14;12:753202. doi: 10.3389/fmicb.2021.753202 (PMC8551967; doi:10.3389/fmicb.2021.753202)
Supplement: Supplementary Table 4 — FunCat Categories assigned to down-regulated genes at 3 h post treatment. Categories were ranked based on the adjusted p-value <0.05 calculated with the Fisher’s enrichment exact test. The percentage of genes assigned to a specific category was calculated with respect to the total number of genes assigned to all the categories identified. [file Table_4.DOCX]

**Table S4.** FunCat Categories assigned to down-regulated genes at 3h post treatment. Categories were ranked based on the adjusted p-value < 0.05 calculated with the Fisher’s enrichment exact test. The percentage of genes assigned to a specific category was calculated with respect to the total number of genes assigned to all the categories identified.

|  | | | | |
| --- | --- | --- | --- | --- |
| **FunCat description** | **FunCat main category** | **Adjusted p-value** | **# genes / category** | **Assigned genes %** |
| transport facilities | Cellular transport, transport facilitation and transport routes | 0.01944061 | 33 / 398 | 80.49 |
| antibiotic resistance | Cell rescue, defense and virulence | 0.01944061 | 4 / 8 | 9.76 |
| glutathione conjugation reaction | Cell rescue, defense and virulence | 0.01944061 | 4 / 8 | 9.76 |
