# Supplementary material for: Transcriptomic and Ultrastructural Analyses of Pyricularia Oryzae Treated With Fungicidal Peptaibol Analogs of Trichoderma Trichogin
Source: Front Microbiol. 2021 Oct 14;12:753202. doi: 10.3389/fmicb.2021.753202 (PMC8551967; doi:10.3389/fmicb.2021.753202)
Supplement: Supplementary Table 5 — Go Categories assigned to up-regulated genes at 3 h post treatment. GO names were ranked based on the adjusted p-value <0.05 calculated with the Fisher’s enrichment exact test. The percentage of genes assigned to a specific GO name was calculated with respect to the total number of genes assigned to all the categories identified. BP = Biological Process; CC = Cellular Component; MF = Molecular function. [file Table_5.DOCX]

**Table S5.** Go Categories assigned to up-regulated genes at 3h post treatment. GO names were ranked based on the adjusted p-value < 0.05 calculated with the Fisher’s enrichment exact test. The percentage of genes assigned to a specific GO name was calculated with respect to the total number of genes assigned to all the categories identified. BP = Biological Process; CC = Cellular Component; MF = Molecular function.

|  | | | | |
| --- | --- | --- | --- | --- |
| **GO term** | **GO term type** | **Adjusted p-value** | **# genes / category** | **Assigned genes %** |
| protein transport | BP | 3.51981E-05 | 24 / 82 | 5.58 |
| vesicle-mediated transport | BP | 0.000133176 | 17 / 50 | 3.95 |
| membrane | CC | 0.000133176 | 86 / 594 | 20.00 |
| proteasome complex | CC | 0.000133176 | 12 / 27 | 2.79 |
| cytoplasmic vesicle | CC | 0.000141435 | 8 / 12 | 1.86 |
| endoplasmic reticulum | CC | 0.000216281 | 20 / 73 | 4.65 |
| calcium ion transport | BP | 0.000216281 | 6 / 7 | 1.40 |
| calcium ion transmembrane transport | BP | 0.000216281 | 6 / 7 | 1.40 |
| Golgi apparatus | CC | 0.000484312 | 11 / 27 | 2.56 |
| cellular nitrogen compound biosynthetic process | BP | 0.000545076 | 14 / 43 | 3.26 |
| Golgi membrane | CC | 0.000660003 | 8 / 15 | 1.86 |
| intracellular protein transport | BP | 0.001341954 | 17 / 65 | 3.95 |
| COPI vesicle coat | CC | 0.002781353 | 4 / 4 | 0.93 |
| cation transport | BP | 0.004347362 | 13 / 46 | 3.02 |
| structural molecule activity | MF | 0.005277233 | 7 / 15 | 1.63 |
| phosphatidylinositol dephosphorylation | BP | 0.009353184 | 4 / 5 | 0.93 |
| integral component of endoplasmic reticulum membrane | CC | 0.009353184 | 4 / 5 | 0.93 |
| nitronate monooxygenase activity | MF | 0.009353184 | 4 / 5 | 0.93 |
| integral component of membrane | CC | 0.0137884 | 86 / 700 | 20.00 |
| peptidase activity | MF | 0.0137884 | 22 / 118 | 5.12 |
| 3-oxoacyl-[acyl-carrier-protein] synthase activity | MF | 0.01680825 | 3 / 3 | 0.70 |
| signal peptidase complex | CC | 0.01680825 | 3 / 3 | 0.70 |
| protein retention in ER lumen | BP | 0.01680825 | 3 / 3 | 0.70 |
| melanin biosynthetic process | BP | 0.01680825 | 3 / 3 | 0.70 |
| dolichyl-diphosphooligosaccharide-protein glycotransferase activity | MF | 0.01680825 | 3 / 3 | 0.70 |
| endoplasmic reticulum membrane | CC | 0.02069002 | 8 / 25 | 1.86 |
| actin binding | MF | 0.02069002 | 8 / 25 | 1.86 |
| ER to Golgi vesicle-mediated transport | BP | 0.04378232 | 7 / 22 | 1.63 |
| calcium-transporting ATPase activity | MF | 0.04913599 | 3 / 4 | 0.70 |
| ARF guanyl-nucleotide exchange factor activity | MF | 0.04913599 | 3 / 4 | 0.70 |
| regulation of ARF protein signal transduction | BP | 0.04913599 | 3 / 4 | 0.70 |
| signal peptide processing | BP | 0.04913599 | 3 / 4 | 0.70 |
| cation-transporting ATPase activity | MF | 0.04947875 | 7 / 23 | 1.63 |
