# Supplementary material for: Transcriptomic and Ultrastructural Analyses of Pyricularia Oryzae Treated With Fungicidal Peptaibol Analogs of Trichoderma Trichogin
Source: Front Microbiol. 2021 Oct 14;12:753202. doi: 10.3389/fmicb.2021.753202 (PMC8551967; doi:10.3389/fmicb.2021.753202)
Supplement: Supplementary Table 6 — Go Categories assigned to down-regulated genes at 3 h post treatment. GO names were ranked based on the adjusted p-value <0.05 calculated with the Fisher’s enrichment exact test. The percentage of genes assigned to a specific GO name was calculated with respect to the total number of genes assigned to all the categories identified. BP = Biological Process; CC = Cellular Component; MF = Molecular function. [file Table_6.DOCX]

**Table S6.** Go Categories assigned to down-regulated genes at 3h post treatment. GO names were ranked based on the adjusted p-value < 0.05 calculated with the Fisher’s enrichment exact test. The percentage of genes assigned to a specific GO name was calculated with respect to the total number of genes assigned to all the categories identified. BP = Biological Process; CC = Cellular Component; MF = Molecular function.

|  | | | | |
| --- | --- | --- | --- | --- |
| **GO term** | **GO term type** | **Adjusted p-value** | **# genes / category** | **Assigned genes %** |
| mycelium development | BP | 2.95471E-10 | 62 / 623 | 22.96 |
| oxidoreductase activity | MF | 1.51779E-07 | 59 / 680 | 21.85 |
| oxidation-reduction process | BP | 0.0000111 | 64 / 868 | 23.70 |
| transmembrane transport | BP | 0.002217 | 34 / 426 | 12.59 |
| integral component of membrane | CC | 0.004611 | 47 / 700 | 17.41 |
| 2 iron, 2 sulfur cluster binding | MF | 0.039902 | 4/12 | 1.48 |
