# Supplementary material for: Transcriptomic and Ultrastructural Analyses of Pyricularia Oryzae Treated With Fungicidal Peptaibol Analogs of Trichoderma Trichogin
Source: Front Microbiol. 2021 Oct 14;12:753202. doi: 10.3389/fmicb.2021.753202 (PMC8551967; doi:10.3389/fmicb.2021.753202)
Supplement: Supplementary Table 7 — Pyricularia oryzae genes up-regulated after 3 h from peptide treatment. [file Table_7.DOCX]

**Table S7.** *Pyricularia oryzae* genes up-regulated after 3 hours from peptide treatment.

| Genes up-regulated at 3 h after peptide treatment | | | | | | | | | |
| --- | --- | --- | --- | --- | --- | --- | --- | --- | --- |
| Cytochrome P450 | MGG_06872 | MGG_08494 | MGG_09945 | MGG_00832 | MGG_01353 | MGG_03392 | MGG_17084 | MGG_01925 | MGG_12479 |
| NADH-cytochrome B5 reductase | MGG_07307 |  |  |  |  |  |  |  |  |
| Pisatin demetyilase | MGG_04404 |  |  |  |  |  |  |  |  |
| NADP/NADPH oxidoreductase | MGG_16813 | MGG_09785 | MGG_01569 | MGG_00769 |  |  |  |  |  |
| NADH oxidoreductase | MGG_08297 |  |  |  |  |  |  |  |  |
| Oxidoreductase domain | MGG_05346 | MGG_17816 | MGG_07009 |  |  |  |  |  |  |
| Oxygenase superfamily | MGG_00953 |  |  |  |  |  |  |  |  |
| Glucose-methanol-choline oxidoreductase | MGG_08072 |  |  |  |  |  |  |  |  |
| Thioredoxin | MGG_13208 |  |  |  |  |  |  |  |  |
|  |  |  |  |  |  |  |  |  |  |
| Glucose 6-P dehydrogenase | MGG_09926 |  |  |  |  |  |  |  |  |
| Glutamate cysteine ligase | MGG_01345 |  |  |  |  |  |  |  |  |
|  |  |  |  |  |  |  |  |  |  |
| Glutathione S transferase / drug detoxification | MGG_05367 | MGG_01410 | MGG_15837 |  |  |  |  |  |  |
| Glutathione-dependent formaldehyde-activating enzyme | MGG_16408 | MGG_08201 |  |  |  |  |  |  |  |
| Glutathione reductase | MGG_12749 |  |  |  |  |  |  |  |  |
| Gamma glutamyl transferase | MGG_03882 |  |  |  |  |  |  |  |  |
| Gamma glutamylcyclo transferase | MGG_04817 |  |  |  |  |  |  |  |  |
|  |  |  |  |  |  |  |  |  |  |
| Galactose oxidase | MGG_07302 | MGG_14917 | MGG_02582 |  |  |  |  |  |  |
| Isoamylalchool oxidase | MGG_02818 |  |  |  |  |  |  |  |  |
| SOD | MGG_13177 |  |  |  |  |  |  |  |  |
| Cu radical oxidase | MGG_01655 |  |  |  |  |  |  |  |  |
| Peroxidase domain | MGG_08200 |  |  |  |  |  |  |  |  |
|  |  |  |  |  |  |  |  |  |  |
| Response to oxidative stress / metabolize drugs | MGG_09400 |  |  |  |  |  |  |  |  |
| Phosphatidyl inositol transfer protein | MGG_00871 |  |  |  |  |  |  |  |  |
| Flavin binding monoxygenase | MGG_04751 |  |  |  |  |  |  |  |  |
| N-acetyl transferase ATS1 | MGG_09867 |  |  |  |  |  |  |  |  |
| Endo-glucanase | MGG_14602 | MGG_07686 | MGG_09709 | MGG_00319 |  |  |  |  |  |
|  |  |  |  |  |  |  |  |  |  |
| ABC transporter | MGG_11754 | MGG_09941 | MGG_11547 | MGG_08918 | MGG_17054 |  |  |  |  |
| Leptomycin resistance protein (ABC multidrug transporter) | MGG_00141 |  |  |  |  |  |  |  |  |
|  |  |  |  |  |  |  |  |  |  |
| OPT oligopeptide transporter | MGG_07228 | MGG_10200 | MGG_11429 |  |  |  |  |  |  |
|  |  |  |  |  |  |  |  |  |  |
| Autophagy-related proteins ATG17 – ATG3 – ATG9 | MGG_07667 | MGG_17909 | MGG_09559 |  |  |  |  |  |  |
| Authophagy Ubiquitin-like ATG7 | MGG_07297 |  |  |  |  |  |  |  |  |
| Cys protease ATG4 (authophagy related protein) | MGG_03580 |  |  |  |  |  |  |  |  |
| NACHT domain protein | MGG_05125 | MGG_00388 |  |  |  |  |  |  |  |
| Caffeine induced death protein | MGG_11129 |  |  |  |  |  |  |  |  |
| Heterokaryon incompatibility protein (HET) | MGG_07794 | MGG_08495 | MGG_15242 | MGG_16825 | MGG_00082 | MGG_00569 | MGG_05985 |  |  |
|  |  |  |  |  |  |  |  |  |  |
| 26S proteaseome regulatory subunit | MGG_02608 | MGG_06504 | MGG_01581 | MGG_03524 | MGG_04506 |  |  |  |  |
| Proteaseome subunit | MGG_05991 | MGG_07165 | MGG_15540 | MGG_00331 | MGG_00170 |  |  |  |  |
| Proteaseome activator | MGG_04342 |  |  |  |  |  |  |  |  |
| Proteasome conponent | MGG_04529 |  |  |  |  |  |  |  |  |
| Proteasome endopeptidase complex | MGG_04053 |  |  |  |  |  |  |  |  |
| Ubiquitin conjugated enzyme | MGG_01807 |  |  |  |  |  |  |  |  |
| Ubiquitin C-terminal hydrolase | MGG_02970 |  |  |  |  |  |  |  |  |
| Cullin | MGG_07145 |  |  |  |  |  |  |  |  |
| Ubiquitin hydrolase (cys peptidase) | MGG_06319 |  |  |  |  |  |  |  |  |
|  |  |  |  |  |  |  |  |  |  |
| Hsp70 chaperone (dnaJ) | MGG_07502 | MGG_07156 | MGG_02503 | MGG_03039 | MGG_07716 |  |  |  |  |
|  |  |  |  |  |  |  |  |  |  |
| Maintenance of mytochondria morphology | MGG_08853 |  |  |  |  |  |  |  |  |
|  |  |  |  |  |  |  |  |  |  |
| DNA repair & recombination process | MGG_11047 | MGG_04405 | MGG_17052 | MGG_03132 |  |  |  |  |  |
| Chromatin structure remodeling & maintenance | MGG-11643 | MGG_03487 |  |  |  |  |  |  |  |
| General transcription factor & DNA repair | MGG_15089 |  |  |  |  |  |  |  |  |
| CENP-S protein | MGG_15064 |  |  |  |  |  |  |  |  |
| HMG box (chromatin remodeling) | MGG_00136 |  |  |  |  |  |  |  |  |
| DNA mismatch repair protein | MGG_00704 |  |  |  |  |  |  |  |  |
|  |  |  |  |  |  |  |  |  |  |
| Melanin biosynthesis | MGG_05059 | MGG_02252 | MGG_07216 | MGG_07219 | MGG_07613 |  |  |  |  |
| Laccase | MGG_13464 | MGG_11608 |  |  |  |  |  |  |  |
|  |  |  |  |  |  |  |  |  |  |
| Chitin synthase | MGG_01802 | MGG_09551 |  |  |  |  |  |  |  |
| Chitin deacetylase | MGG_08774 |  |  |  |  |  |  |  |  |
| Glucosammine 6P N-acetyl transferase | MGG_10574 |  |  |  |  |  |  |  |  |
| N-acetyl glucosammine deacetylase | MGG_00620 |  |  |  |  |  |  |  |  |
| Chitin recognition protein/deacetylase | MGG_14966 |  |  |  |  |  |  |  |  |
| Alpha1,2 mannosidase | MGG_00695 |  |  |  |  |  |  |  |  |
| Phosphatidyl inositol N-acetylglucosammine transferase | MGG_03716 |  |  |  |  |  |  |  |  |
| N-acetylglucosammine utilization | MGG_00625 |  |  |  |  |  |  |  |  |
| N-acetylglucosammine transporter | MGG_16866 |  |  |  |  |  |  |  |  |
| LysM domain protein (chitin binding) | MGG_07571 | MGG_01502 |  |  |  |  |  |  |  |
|  |  |  |  |  |  |  |  |  |  |
| 1,3 beta-glucan synthase | MGG_00865 |  |  |  |  |  |  |  |  |
| Glucanase | MGG_07306 | MGG_07686 | MGG_09709 |  |  |  |  |  |  |
| Endo beta 1,6 glucanase | MGG_14602 |  |  |  |  |  |  |  |  |
| Glucan 1,3 beta glucosidase | MGG_09995 | MGG_06512 |  |  |  |  |  |  |  |
| 1,3 beta glucanosyl transferase | MGG_08370 | MGG_08370 |  |  |  |  |  |  |  |
|  |  |  |  |  |  |  |  |  |  |
| Glycosyl phosphatidyl inositol anchored membrane | MGG_04599 |  |  |  |  |  |  |  |  |
|  |  |  |  |  |  |  |  |  |  |
| MARVEL protein (membrane apposition events) | MGG_08113 |  |  |  |  |  |  |  |  |
| CRIB domain | MGG_06449 |  |  |  |  |  |  |  |  |
| GRAM domain protein | MGG_11211 |  |  |  |  |  |  |  |  |
| Fatty acid synthase S-acetyl transferase | MGG_12214 |  |  |  |  |  |  |  |  |
| Fatty acid synthase | MGG_12154 | MGG_04118 |  |  |  |  |  |  |  |
| PKS-NRPS synthetase TAS1 | MGG_07803 |  |  |  |  |  |  |  |  |
| Linoleate diol synthase | MGG_13239 |  |  |  |  |  |  |  |  |
| Phosphatidyl serine decarboxylase | MGG_07037 |  |  |  |  |  |  |  |  |
| Lysophospholipase | MGG_07287 |  |  |  |  |  |  |  |  |
| Lipase | MGG_06610 |  |  |  |  |  |  |  |  |
| GDSL-lipase | MGG_05879 |  |  |  |  |  |  |  |  |
|  |  |  |  |  |  |  |  |  |  |
| Integral membrane proteins | MGG_08003 | MGG_05121 | MGG_08252 | MGG_02410 |  |  |  |  |  |
|  |  |  |  |  |  |  |  |  |  |
| Calpain Cys protease | MGG_14872 | MGG_07573 | MGG_15810 |  |  |  |  |  |  |
| Metalloprotease | MGG_10104 | MGG_03817 | MGG_04858 |  |  |  |  |  |  |
| Peptidase S9 | MGG_09249 |  |  |  |  |  |  |  |  |
| Ser aminopeptidase | MGG_08589 |  |  |  |  |  |  |  |  |
| Leu aminopeptidase | MGG_10171 |  |  |  |  |  |  |  |  |
| Ser carboxypapetidase | MGG_00775 | MGG_15423 |  |  |  |  |  |  |  |
| Aspartyl protease | MGG_11945 |  |  |  |  |  |  |  |  |
| Zinc protease | MGG_16395 |  |  |  |  |  |  |  |  |
| Subtilase family serinprotease | MGG_02863 |  |  |  |  |  |  |  |  |
| Dipeptidyl peptidase | MGG_07745 | MGG_07877 |  |  |  |  |  |  |  |
| Peptide hydrolase | MGG_06587 |  |  |  |  |  |  |  |  |
| Vacuolar amino-(metallo)peptidase | MGG_07536 | MGG_02834 |  |  |  |  |  |  |  |
| TAP-like protein similar to tripeptidyl aminopeptidase | MGG_00531 |  |  |  |  |  |  |  |  |
| CAAX prenyl protease | MGG_06951 |  |  |  |  |  |  |  |  |
|  |  |  |  |  |  |  |  |  |  |
| Met permease | MGG_03103 |  |  |  |  |  |  |  |  |
| aa permease/transporter | MGG_06036 | MGG_07606 |  |  |  |  |  |  |  |
| Allantoate permease | MGG_10328 |  |  |  |  |  |  |  |  |
|  |  |  |  |  |  |  |  |  |  |
| Pyriculariol toxin biosynthesis | MGG_10910 | MGG_12983 | MGG_16812 |  |  |  |  |  |  |
| Aflatoxin efflux pump (transmembrane transporter) | MGG_01778 |  |  |  |  |  |  |  |  |
| Trichitecene 3-O acetyltransferase (detox) | MGG_08440 |  |  |  |  |  |  |  |  |
| Beta-lactamase (multiresistance to antibiotic) | MGG_08486 |  |  |  |  |  |  |  |  |
|  |  |  |  |  |  |  |  |  |  |
| Ca2+ transport ATPase | MGG_04890 | MGG_02487 | MGG_04550 |  |  |  |  |  |  |
| Vacuolar Ca2+ trasporter | MGG_11454 | MGG_13224 | MGG_11454 |  |  |  |  |  |  |
| K+/Na+ efflux | MGG_02074 | MGG_13279 |  |  |  |  |  |  |  |
| K+ trasporter | MGG_09119 | MGG_07661 |  |  |  |  |  |  |  |
| Na+/K+ exporter | MGG_06437 |  |  |  |  |  |  |  |  |
|  |  |  |  |  |  |  |  |  |  |
| Salicylate hydroxylase (SA degradation) | MGG_08293 |  |  |  |  |  |  |  |  |
| Salicylaldheide dehydrogenase (SA synthesis) | MGG_00652 |  |  |  |  |  |  |  |  |
