# Supplementary material for: Transcriptomic and Ultrastructural Analyses of Pyricularia Oryzae Treated With Fungicidal Peptaibol Analogs of Trichoderma Trichogin
Source: Front Microbiol. 2021 Oct 14;12:753202. doi: 10.3389/fmicb.2021.753202 (PMC8551967; doi:10.3389/fmicb.2021.753202)
Supplement: Supplementary Table 8 — Pyricularia oryzae genes down-regulated after 3 h from peptide treatment. [file Table_8.DOCX]

**Table S8.** *Pyricularia oryzae* genes down-regulated after 3 hours from peptide treatment.

| Genes down-regulated at 3 h after peptide treatment | | | | | |
| --- | --- | --- | --- | --- | --- |
| Cytochrome P450 | MGG_02329 | MGG_08498 | MGG_05215 | MGG_13768 | MGG_17453 |
| Cytochrome b245 | MGG_00750 | MGG_06559 |  |  |  |
| Cytochrome b5/oxidoreductase | MGG_00861 | MGG_06289 |  |  |  |
| Glucose-methanol-choline oxidoreductase | MGG_16853 |  |  |  |  |
| NAD/NADPH oxidoreductase | MGG_02337 | MGG_04736 |  |  |  |
| NADP/NADPH deydrogenase | MGG_04569 | MGG_00220 |  |  |  |
| FAD oxidoreductase | MGG_14068 |  |  |  |  |
| Oxygen oxidoreductase | MGG_09725 |  |  |  |  |
| NADH oxydase | MGG_03823 |  |  |  |  |
|  |  |  |  |  |  |
| Galactose oxidase | MGG_02368 |  |  |  |  |
| PKS | MGG_14897 |  |  |  |  |
| Catalase | MGG_06442 |  |  |  |  |
|  |  |  |  |  |  |
| Glutathione peroxidase | MGG_07460 |  |  |  |  |
| Glutathione S transferase | MGG_12569 | MGG_06747 |  |  |  |
| Glutathione dependent formaldehyde activating enzyme | MGG_07961 |  |  |  |  |
| S formyl glutathione hydrolase | MGG_05317 |  |  |  |  |
| Membrane protein involved in glutathione metabolism | MGG_06907 |  |  |  |  |
| 5-oxoprolinase | MGG_00771 |  |  |  |  |
|  |  |  |  |  |  |
| Ecdysteroid kinase (detoxification?) | MGG_09503 |  |  |  |  |
| Betaine aldehyde dehydrogenase ° | MGG_03263 |  |  |  |  |
|  |  |  |  |  |  |
| Multidrug and toxin extrusion protein | MGG_04182 |  |  |  |  |
| Stress induced phosphoprotein | MGG_08980 |  |  |  |  |
|  |  |  |  |  |  |
| Authophagy related protein ATG27 / mannose-6-phosphate receptor | MGG_09271 |  |  |  |  |
|  |  |  |  |  |  |
| DNA repair protein | MGG_07015 |  |  |  |  |
| G/U uracyl DNA glycosidase (repair DNA) | MGG_11518 |  |  |  |  |
| DNA repair helicase | MGG_06470 |  |  |  |  |
| Chromo-domain | MGG_17583 |  |  |  |  |
| Thiamine thazole synthase | MGG_03098 |  |  |  |  |
| RECA2/RAD1 | MGG_01470 |  |  |  |  |
|  |  |  |  |  |  |
| Hsp78 | MGG_04437 |  |  |  |  |
| Hsp | MGG_05719 | MGG_03329 | MGG-06459 |  |  |
| Hsp70 (pH stress) | MGG_06958 | MGG_03983 |  |  |  |
| SIS1 hsp40 DNAj chaperone | MGG_08180 |  |  |  |  |
|  |  |  |  |  |  |
| Tyrosinase (copper monoxygenase) | MGG_07419 | MGG_06169 |  |  |  |
| Laccase1 | MGG_08523 |  |  |  |  |
|  |  |  |  |  |  |
| Exo beta1,3-1,6 glucan hydrolase | MGG_13993 |  |  |  |  |
| Beta-glucanase | MGG_06493 |  |  |  |  |
| 1,3 beta glucanosyl transferase | MGG_03208 |  |  |  |  |
|  |  |  |  |  |  |
| Chitin binding protein | MGG_17153 |  |  |  |  |
| Acidic chitinase | MGG_04732 |  |  |  |  |
| Polysaccharide deacetylase | MGG_01922 |  |  |  |  |
|  |  |  |  |  |  |
| Acyltransferase | MGG_00343 |  |  |  |  |
| MARVEL protein (membrane apposition events) | MGG_08535 | MGG_08055 |  |  |  |
| Proteolipid membrane potential modulator | MGG_10723 |  |  |  |  |
| Lipid phosphate phospathase | MGG_05988 |  |  |  |  |
| Glycerol oxidase | MGG_01281 |  |  |  |  |
| Triacylglycerol lipase FGL2 | MGG_04555 |  |  |  |  |
| Lipase | MGG_09839 | MGG_14057 |  |  |  |
| GDSL-lipase | MGG_15479 | MGG_10441 |  |  |  |
|  |  |  |  |  |  |
| Isocytrate lyase | MGG_04895 |  |  |  |  |
|  |  |  |  |  |  |
| Endoprotease | MGG_02275 |  |  |  |  |
| Carboxypeptidase | MGG_02309 | MGG_07850 | MGG_13765 | MGG_10330 |  |
| Extracellular protease | MGG_02531 |  |  |  |  |
| Metallo endopeptidase | MGG_02529 |  |  |  |  |
| Intracellular Cys peptidase | MGG_01679 |  |  |  |  |
| Aspartyl protease | MGG_07179 |  |  |  |  |
| Cys protease mitochondrial | MGG_02695 |  |  |  |  |
|  |  |  |  |  |  |
| MFS protein / Pyriculariol biosynthesis | MGG_04850 |  |  |  |  |
| Ustiloxin toxin biosynthesis | MGG_00272 |  |  |  |  |
| Snodprot1 | MGG_05344 |  |  |  |  |
| Necrosis ethylene inducing protein | MGG_08454 | MGG_10532 |  |  |  |
| Cercosporin toxin biosynthesis | MGG_00427 |  |  |  |  |
|  |  |  |  |  |  |
| aa permease | MGG_13963 |  |  |  |  |
| Lys permease | MGG_08129 |  |  |  |  |
| Pro permease | MGG_04216 |  |  |  |  |
| Aminotransferase | MGG_03494 | MGG_10969 |  |  |  |
| aa transporter | MGG_08827 | MGG_07639 |  |  |  |
| aa biosynthesis | MGG_03889 |  |  |  |  |
| Allantoate permease | MGG_04099 |  |  |  |  |
|  |  |  |  |  |  |
| Spore development regulator | MGG_00617 |  |  |  |  |
| Glutathione dehydrogenase | MGG_06011 |  |  |  |  |
